# Using an agent-based model to evaluate the effect of producer specialization on the epidemiological resilience of livestock production networks

**DOI:** 10.1371/journal.pone.0194013

**Published:** 2018-03-09

**Authors:** Serge W. Wiltshire

**Affiliations:** Food Systems, University of Vermont, Burlington, VT, United States of America; Universidad Rey Juan Carlos, SPAIN

## Abstract

An agent-based computer model that builds representative regional U.S. hog production networks was developed and employed to assess the potential impact of the ongoing trend towards increased producer specialization upon network-level resilience to catastrophic disease outbreaks. Empirical analyses suggest that the spatial distribution and connectivity patterns of contact networks often predict epidemic spreading dynamics. Our model heuristically generates realistic systems composed of hog producer, feed mill, and slaughter plant agents. Network edges are added during each run as agents exchange livestock and feed. The heuristics governing agents’ contact patterns account for factors including their industry roles, physical proximities, and the age of their livestock. In each run, an infection is introduced, and may spread according to probabilities associated with the various modes of contact. For each of three treatments—defined by one-phase, two-phase, and three-phase production systems—a parameter variation experiment examines the impact of the spatial density of producer agents in the system upon the length and size of disease outbreaks. Resulting data show phase transitions whereby, above some density threshold, systemic outbreaks become possible, echoing findings from percolation theory. Data analysis reveals that multi-phase production systems are vulnerable to catastrophic outbreaks at lower spatial densities, have more abrupt percolation transitions, and are characterized by less-predictable outbreak scales and durations. Key differences in network-level metrics shed light on these results, suggesting that the absence of potentially-bridging producer–producer edges may be largely responsible for the superior disease resilience of single-phase “farrow to finish” production systems.

## Introduction

There is widespread agreement among livestock veterinarians and epidemiologists that mitigating disease outbreaks is critical to promote food safety, maintain food availability, and reduce economic risk in the marketplace. Livestock epidemiologists commonly focus on promoting the adoption of discrete biosecurity measures, such as truck wash facilities and biocontainment procedures at individual premises [1]. However, empirical research has increasingly revealed the importance of understanding how the structures of trade and transportation networks can aid in predicting and preventing outbreaks [2–4]. In light of these observations, more work is clearly needed to understand how livestock biosecurity may be bolstered from a systems perspective.

Recent years have seen significant structural changes within the U.S. hog industry, with a marked trend toward increased producer specialization. Whereas in the past it was standard practice for a single producer to take a pig “from farrow to finish,” it is now increasingly common for livestock to be housed at two or, more recently, three different producer operations throughout their lives, with each operation specializing in a specific life cycle stage [5]. While operational efficiency advantages may be gained through increased specialization, its effect on disease spread is not fully understood.

To address this question, we developed an agent-based susceptible / infective (SI) computer model to simulate epidemiological events in hog production networks. Agent-based models (ABMs) have been used extensively to analyze complex phenomena that emerge from the relatively-simple actions of a cohort of interacting individuals [6–8]. Using the 2013 PEDv outbreak as a case study, three key mechanisms facilitating disease transmission in the hog industry were identified: (a) the transfer of infected animals between premises, (b) deliveries of contaminated feed, and (c) contaminated livestock and feed transportation equipment [9, 10]. Contact between producers, slaughter plants, and feed mills was found to be largely responsible for spreading the virus. In our ABM, structured populations of these three agent types are placed in the simulation, an infection is introduced randomly, and decision heuristics define how and when agents come into contact, potentially transmitting the infection. Using this model, we report on a series of parameter variation experiments that investigate the epidemic spread characteristics resulting from varied levels of producer specialization and numbers of producers in the system, finding evidence of percolation dynamics, with increased specialization leading to significantly diminished epidemiological resilience.

### Percolation theory

The “robust yet fragile” nature that describes a diversity of complex systems offers a useful framework to understand the spread of diseases through networks. Pathogens are regularly introduced with little consequence, but due to stochasticity and internal heterogeneities in contact network structures, a single pathogen can occasionally ignite a widespread epidemic [11–16]. Percolation is the mathematical phase-change that occurs when the density of entities in a system becomes sufficient that the expected outbreak magnitude no longer scales linearly with each added node, but instead accelerates rapidly toward near-complete spreading. The point at which this transition occurs—the percolation threshold—is defined as the density at which, in an infinite network, the expected size of the “giant component” is also infinite [17–22]. While much of the work in this area has concerned itself with analytically-formalizing percolation behavior in relatively-simple systems, the insights gained through such investigations are relevant for understanding dynamical regimes in complex real-world systems as well. To investigate percolation in our experimental results, we numerically assess how the size and duration of epidemic events in a series of model runs scale with the addition of producer nodes.

### Models of epidemic spreading

At the core of many model-based inquiries into disease spread is the susceptible / infective / recovered (SIR) framework. In SIR models, an infective individual may spread a disease to susceptible individuals for a period of time, after which the infective individual transitions to the recovered state and cannot be reinfected [23]. The SI framework, a common SIR derivative, allows for repeated infections. SIR models based on differential equations (DEs) have successfully replicated many of the complex temporal patterns typical of epidemics [24–26]. However, the DE approach has been criticized, as it implicitly posits both a homogeneous population and complete mixing [27]. Structured population models partially overcome these shortfalls, defining heterogeneous distributions for parameters such as age, size, spatial position, and movement [28, 29]. Cellular Automata (CA) SIR models additionally reproduce spatial phenomena such as waves of infection radiating outward from a source [30, 31], and can also produce percolation-type phase transitions [32], providing insights into the impact of agents’ relative spatial positions on spreading dynamics [33, 34]. More advanced CA models examine the impact of heterogeneous susceptibilities, transmission rates, and infectious periods [35], as well as modulating parameters as a function of spatial proximity [36, 37].

In recent years, epidemiological modelers have increasingly investigated the role of the structural topology of “mixing networks” on disease percolation [38]. SIR simulation studies on complex networks demonstrate the impact of degree distribution on the speed, size, and variability of epidemic events, with more heterogeneously-distributed networks pushing the percolation threshold towards zero as *N* → ∞ [39]. Epidemics on scale-free networks cascade from highly-connected hubs through smaller degree classes [40], although a sharp percolation threshold is not observed [41, 42]. In metapopulation (or subpopulation) networks, surpassing critical values for the rate of spreading between subpopulations can trigger percolation, shedding light on the mechanism behind the “robust yet fragile” nature of these systems [43–45].

Coelho, Cruz, and Codeço [46] characterize the complexification of epidemiological simulations over time as a shift between “strategic models” that explore the fundamental features of epidemics, to “tactical models” that mirror the conditions within which a real-world epidemic may unfold. Agent-based models are often examples of the latter, generating empirically-calibrated networks of interacting agents that are heterogeneous not only in their parameter values, but also in the behavioral heuristics that govern how and when contact occurs [47]. Modelers can hard-code agents’ positions or spatial distributions using a GIS framework [48], and may incorporate empirical data—such as airline routes or telephone records [49, 50]; or, in the case of livestock epidemics, the operational details and locations of farms [51, 52]—that have been shown to correlate with outbreak patterns. Other modelers let networks emerge organically during each model run as a result of agents’ decision-making heuristics. Using the latter methodology, Ghani & Garnett [53] found network centrality measures that predicted an agent’s chance of either getting or spreading a sexually-transmitted disease. Eubank et al. [54] developed an ABM that utilized heuristics parameterized from large-scale datasets to generate realistic urban social contact networks and identified resulting epidemiological vulnerabilities. Gojovic et al. [55] implemented a model to evaluate optimal immunization strategies during the 2009 H1N1 pandemic, using demographic and employment records to assign agent parameters, and incorporating differential transmission probabilities for multiple contexts. Keeling et al. [56] developed a model of U.K. farms—parameterized via census data—and performed a Monte Carlo simulation to understand how factors including agent heterogeneities and movement restrictions explain the observed spread of the 2001 UK Foot and Mouth epidemic. The ABM we have developed for this experiment builds on prior work in this area, leveraging empirical data to heuristically generate hog production systems that are structurally-parallel to real-world examples, and encoding behavioral rules in collaboration with industry experts that allow the contact networks underlying disease spread to emerge organically in each model run.

### Network analytics and epidemiological vulnerability

Bailey [43] was among the first to suggest that, while contagions are generally confined to small network clusters, global epidemics may result when an edge forms a bridge between clusters. This pattern has been empirically observed in both human and livestock epidemics. Firestone et al. [2] analyzed infected premises during the 2007 Equine Influenza outbreak in Australia, finding strong evidence that the movement of infected horses between spatially-clustered groups of premises correlated with the spread of the disease. Fournié et al. [3] investigated the network connectivity patterns of agents involved in Vietnam’s live bird markets during an H5N1 influenza outbreak, concluding that the contact network could have been largely “disconnected” by focusing on disinfection of transportation equipment at a few of the large hubs. The structure of transportation networks for feed and livestock was found to be the primary factor underlying the 2013 U.S. hog industry PEDv epidemic, with slaughter plants and feed mills serving as the primary hubs [4].

Network theorists have conducted several investigations into the relationships between metrics describing a network’s structure, and the propensity of that network to promote or inhibit spreading [57, 58]. Experiment two below evaluates six network metrics that may impact epidemiological resilience. As a baseline, we investigate whether—as would be expected—hog production networks with higher average degree 〈*k*〉 promote disease spread. Small-world graphs—a class into which many real-world livestock production networks fall [59–61]—are characterized by a smaller mean shortest path length 〈*l*〉 and greater clustering *C* than corresponding random graphs with equivalent 〈*k*〉 [62]. Research shows that spreading in small-world networks (versus corresponding random graphs) proceeds more rapidly but results in fewer infected nodes, and also that small-world networks exhibit significantly higher *k*-core densities [63]. Other studies have found that *k*-core boundaries often define the part of a graph in which a spreading event is more likely to persist [64]. Experiment two thus analyzes the role of *k*-core size *S*_*kc*_, in addition to *k*-core order *O*_*kc*_, and number of *k*-cores *N*_*kc*_. While there is debate over whether weighted versus unweighted—as well as directed versus symmetrized—versions of network metrics are more appropriate, at least in some contexts, metrics calculated on unweighted, symmetrized graphs best predict epidemiological vulnerability [65]. In light of this, we opt to binarize and symmetrize the graphs in our analysis.

### Research questions and hypotheses

This study uses an agent-based model to investigate the impact of two ongoing network-structural trends in the U.S. hog industry upon system-level epidemiological resilience. The first of these trends is the growing spatial density of networks. With more potential trading partners from which to choose, the average degree of the network will tend to increase, which should correlate positively with outbreak severity. The second trend, increasing producer specialization, will necessarily add producer–producer edges where previously there were none. Following the empirical and computational studies cited above, we can hypothesize that a greater probability of large-scale “global” epidemics should be observed wherever the network typology is such that, without these additional edges acting as bridges, the contagion would have been isolated within localized clusters. By systematically varying both the spatial density of the simulated networks, along with the level of producer specialization, the experiments described below investigate how the interplay between these two factors may render a system more or less vulnerable to catastrophic disease outbreaks.

## Materials and methods

### Model design concepts

The Regional U.S. Hog Production Network Biosecurity Model (RUSHPNBM) generates simulated hog production systems composed of producer, slaughter plant, and feed mill agents. Heterogeneous parameter values, multiple interaction contexts, and spatial proximity considerations, are incorporated into agents’ decision heuristics, and determine contact patterns and infection spread potentials. The epidemiological submodel is of the SI type, since, in the case of PEDv, reinfections of the same premises have been reported [66].

RUSHPNBM is a “tactical” model, in that it is empirically-calibrated to mirror a real-world system, but it also aims to avoid being overly context-specific, leaving sufficient flexibility to analyze a variety of scenarios [46]. To this end, elements that were deemed significant facilitators of disease spread by a cohort of industry consultants were included in the model, while many extraneous and/or uncertain details were bracketed [67]. The baseline parameterization—although ground-truthed by our advisors as well as several datasets—is not meant to project the course of a specific infectious agent through any real-world production network, but rather to facilitate a workable and reasonably-realistic simulation useful for understanding the network trends important for this experiment.

The model was developed using AnyLogic 7 software, with all functions written in Java. The sections below provide an overview of initialization procedures, agents’ behavioral heuristics, and parameter calibration methods. For full implementation details, see S1 Protocol.

### Agent initialization

**Fig 1:**
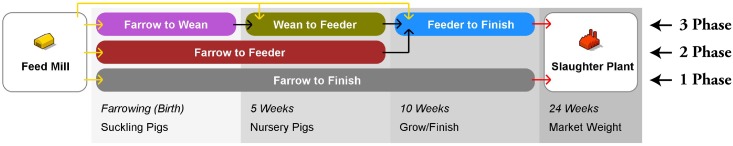
Structure of agent connections in the model. Shows 1-phase (low-specialization), 2-phase (mid-specialization), and 3-phase (high-specialization) connectivity heuristics. Also indicates livestock transfer age conditions.

At model initialization, all agents are assigned a fixed location stochastically within a continuous two-dimensional spatial framework defined by an 880 x 490 unit rectangle, with units representing kilometers. Producer agents are assigned one of five industry roles (see Fig 1) according to distributions corresponding to the treatment scenario (see Experimental design section). The livestock capacity of each producer agent is assigned by drawing from a normal distribution (Table 1 gives parameter values). Producer agents may have one or several batches of pigs, with each batch considered to be the same age. Each producer begins at full capacity, with the age of pig batches drawn from a uniform distribution corresponding to the industry role of the producer.

**Table 1:** Parameter values used in the experiment.

Parameter	Value(s) Explored
*Network parameters*	
Area of network region (km2)	431200
Number of producer agents in the model	[10:10:1500]
Number of livestock per producer [normal distribution; rounded to integer]	*μ* = 1,000; *σ* = 300; *x* ≥ 50
Number of producer production phases	[1, 2, 3]
Number of slaughter plant agents in the model	3
Number of feed mill agents in the model	10
*Epidemiological parameters*	
Suckling pig mortality rate	0.95
Nursery pig mortality rate	0.6
Grow/finish hog mortality rate	0.1
Length of producer infection (days) [triangular distribution]	*μ* = 30; 0 ≤ *x* ≤ 60
Length of slaughter plant contamination (days) [triangular distribution]	*μ* = 5; 0 ≤ *x* ≤ 10
*Farrowing parameters*	
Frequency of farrowing (days)	30
Minimum farrowing quantity as a proportion of producer capacity	0.25
*Producer–producer parameters*	
Maximum producer to producer connection distance (km)	100
Minimum transfer quantity as a proportion of transferee capacity	0.25
Prob. of infection via trailer returning from infected transferee	0.15
*Feed mill–producer parameters*	
Frequency of feed distribution trips (days)	1
Percent of producers in feed mill service area visited per trip	15
Probability that truck will be contaminated upon visiting an infected producer	0.15
Probability that contaminated truck will infect subsequent producers on route	0.15
*Producer–slaughter plant parameters*	
Probability that infected hogs will contaminate slaughter plant receiving area	0.75
Probability of infection via truck returning from infected slaughter plant	0.15

### Network initialization

**Fig 2:**
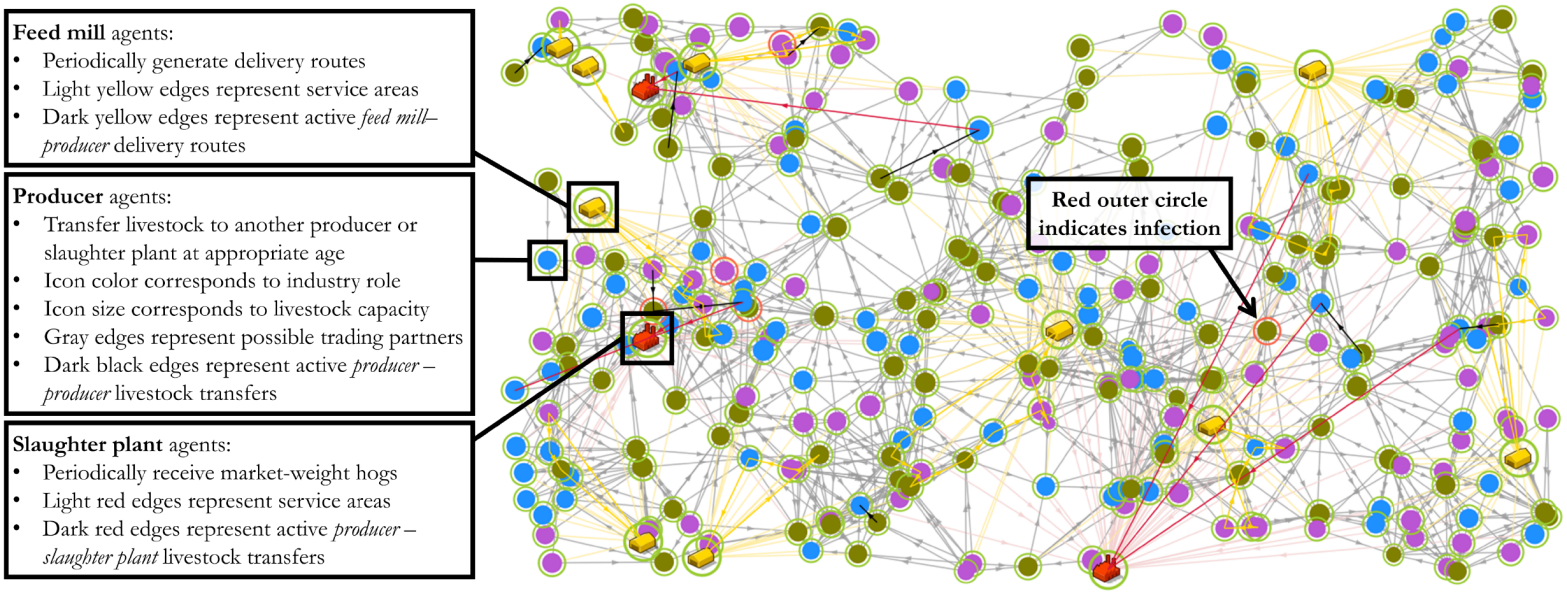
Sample network map as displayed on the model dashboard. Shows agents as nodes and inter-agent contacts (both potential and active) as edges. Key provides an overview of connectivity heuristics for each agent type.

Network edges in the model are subdivided into three contexts: (a) *producer–producer*, (b) *producer–slaughter plant*, and (c) *feed mill–producer*. The basic structure of connections between agents of each industry role is visualized in Fig 1. A network initialization function generates a set of potential trading partners for each agent, defining the possible edges across which contact may occur during the remainder of the model run. Producer agents are each assigned to their most proximal feed mill, and finishing producers are also assigned to their most proximal slaughter plant. Each non-finishing producer is assigned a pool of potential transferee producers of the appropriate industry role for outgoing shipments (see Fig 1), and within a maximum distance of 100km. Fig 2 shows a sample network as displayed on the model dashboard, and briefly describes the heuristics associated with each agent class.

### Initial infection

The initial infection event is triggered after one model year, to skip the transient period and allow the simulation to stabilize. At this point, a single producer agent is selected randomly and transitioned to the infected state.

### Behavioral heuristics

The major functions controlling agent behavior, network connectivity, and infection transmissibility, are detailed below. Specific parameter values appear in Table 1.

#### Farrowing

Farrowing producers (where piglets are born) periodically replenish their inventories by generating new pig batches. New batches are the size of the producer’s spare capacity. A minimum farrowing quantity parameter ensures reasonably-sized batches.

#### Producer–producer livestock transfers

Non-finishing producers transfer pig batches that have reached the transfer age corresponding to their industry role to an appropriate transferee (see Fig 1). A minimum transfer size requirement ensures realistically-sized shipments between producers. Transferee producers are sequentially evaluated in order of proximity until a producer with sufficient excess livestock capacity is identified, at which point the pig batch meeting the transfer age requirement is deleted from the transferring producer’s stock and added to the transferee’s. If the transferring producer is infected, the infected livestock will spread the disease to the transferee. If the transferee producer is infected but the transferring producer is not, the returning “delivery truck” may become contaminated and infect the transferring producer according to a set probability. If a producer becomes infected, the size of each of its pig batches is diminished by the mortality rate associated with the batch’s age. Producers remain infected for a duration determined by a triangular distribution—an intuitive and reliable proxy for the beta distribution [68]—with a mean of 30 days.

#### Producer–slaughter plant livestock transfers

Finishing producers ship livestock to their slaughter plant as soon as a pig batch reaches the designated age. If the transferring producer is infected, the receiving area of the slaughter plant may become contaminated according to a set probability. If infected, a slaughter plant will remain infected for a duration determined by a triangular distribution with a mean of 5 days. If the receiving area of the slaughter plant is already contaminated when a shipment arrives, the returning “delivery truck” may infect the shipping producer according to a set probability.

#### Feed mill–producer delivery routes

Every model day, each feed mill generates a delivery route by first selecting a producer agent within its service area at random. From this location, the nearest producer within the feed mill’s service area that has not been visited becomes the next stop on the route, and this process is repeated until the “delivery truck” has visited the designated number of farms. Should the truck encounter an infected producer premises on its route, the truck may become contaminated according to a set probability. Once a truck is contaminated, the infection may spread to subsequent producers on the route according to a set probability.

### Parameter calibration

The structural makeup and contact patterns of the simulated hog industry network are based on several statistical datasets, as well as qualitative input from a cohort of experts including veterinarians, epidemiologists, and hog industry analysts. Distributions of producer livestock capacity and spatial density were generalized from USDA Census of Agriculture data [69], while slaughter plant density was generalized from USDA Food Safety and Inspection Service data [70]. Feed mill data proved more elusive, so parameterization was based primarily on expert estimates. Temporal aspects of the simulation, such as the frequency with which contact events occur, were generalized from a search of the primary literature coupled with industry expert consultations.

Porcine Epidemic Diarrhea virus (PEDv) is a disease that swept through the U.S. hog industry starting in 2013, causing widespread mortality and morbidity among livestock [71]. This outbreak was used as a case study to calibrate the epidemiological parameters of the model. In consultation with livestock veterinary professionals, reasonable baseline values for parameters such as mortality rates for animals of different ages were chosen. A series of parameter variation experiments were used to hone in on baseline parameter values for infection probabilities and durations such that the infection within the model spread in a manner similar to the patterns observed in the real-world PEDv outbreak, for which tracking data are available [66]. These values were then exogenized as baseline parameters that remained fixed across all experimental runs (Table 1).

### Experimental design

Using the model detailed above, two experiments were performed. The first explored disease percolation by varying the number of producers in the model. The second explored the relationship between key network metrics and epidemiological resilience.

The treatments differed according to the distribution of industry roles assigned to producer agents. Aside from these producer classification assignments, all parameters remained constant across all runs. The treatments are defined as follows (see also Fig 1):

1. High specialization
  - Three-phase production system
  - Equal numbers of Farrow to Wean, Wean to Feeder, and Feeder to Finish producers
2. Medium specialization
  - Two-phase production system
  - Equal numbers of Farrow to Feeder and Feeder to Finish producers
3. Low specialization
  - One-phase production system
  - Farrow to Finish producers only

#### Experiment one

Experiment one was a parameter variation experiment in which, for each treatment scenario, the number of producer agents in the model (*N*_*p*_) was varied between 10 and 1,500 in increments of 10. Since the network region area is fixed, varying *N*_*p*_ corresponds to a change in the spatial density of producers. For each treatment, the model was executed 100 times at each of the 150 *N*_*p*_ values, for a total of 15,000 model runs for each specialization level, or 45,000 runs overall. In each run, the model was stopped 4,135 model days after the initial infection; sufficient time for the infection to either die out or become systemic. Each run generated two dependent variable datapoints: (a) the overall duration of the infection event within the network as a whole, and (b) the proportion of agents that became infected at least once during the model run.

#### Experiment two

In experiment two, features of production networks resulting from differential producer specialization were quantified and analyzed. Contact network data from 150 model runs for each specialization level—with *N*_*p*_ = 100: 100: 1500, and 10 repetitions per parameterization—were exported from the model and analyzed as unweighted, undirected graphs. A similar edge list containing only the subset of nodes that had been infected during each model run was also stored. For both the contact network and the infected component network, six metrics were calculated using functions from the Python NetworkX library [72], with values for each metric plotted against *N*_*p*_ for each treatment. Table 2 gives Python and NetworkX code used in the analyses.

**Table 2:** Python / NetworkX code used in experiment two.

Network Metric	Python / NetworkX Code
Average Degree 〈*k*〉	sum(G.degree().values()) / len(G.nodes())
Average Shortest Path Length 〈*l*〉	nx.average_shortest_path_length(G)
Clustering Coefficient *C*	nx.average_clustering(G)
*k*-core Order *O*_*kc*_	nx.degree(k_core(G), nbunch = k_core(G).nodes()[1])
*k*-core Size *S*_*kc*_	len(nx.k_core(G).nodes())
Number of *k*-cores *N*_*kc*_	nx.number_connected_components(nx.k_core(G))

**Note:** Python 2.7 and NetworkX 2.0 were used for all analyses. The prefix “nx.*” indicates a NetworkX function. “G” represents the NetworkX graph object to be analyzed.

## Results

### Experiment one

**Fig 3:**
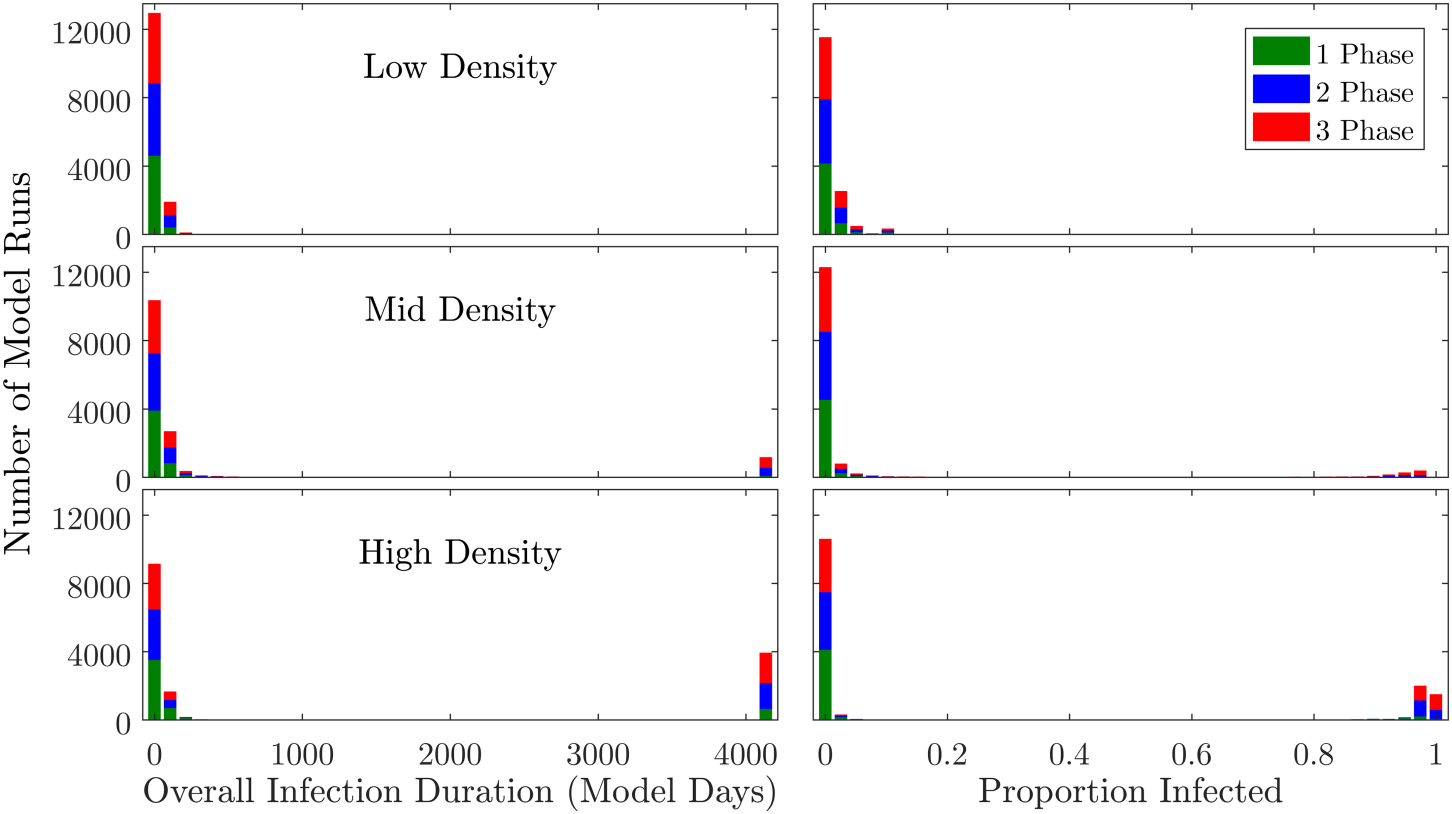
Histograms showing distribution of dependent variables. Infection duration data appear in the left column and proportion of infected agents in the right column, with color indicating producer specialization level. Low density runs were those with 0 < *N*_*p*_ ≤ 500, mid-density 500 < *N*_*p*_ ≤ 1000, and high-density 1000 < *N*_*p*_ ≤ 1500. Data were split into 40 bins.

As an initial step to examine the model output data, histograms were produced to visualize the distribution of outbreak sizes (the proportion of agents infected) and overall infection durations (the time between the initial infection and the last agent recovering from infection, in model days); with data stratified into three *N*_*p*_ ranges (Fig 3). These plots show that the distribution of infection severity—especially in the high-*N*_*p*_ runs—is bimodally-distributed. Kolmogorov-Smirnov normality tests using *N*_*p*_ as the theoretical distribution confirm that, overall, the data are not normally-distributed (for proportion infected *D* = −9.1205, *p* = 0.000; for infection duration *D* = −9.1981, *p* = 0.000). This finding would appear to mirror the literature on epidemic size distributions, suggesting that infection events in the model generally remain within a local cluster, but sometimes explode in scale due to bridging links [2–4, 43–45].

**Fig 4:**
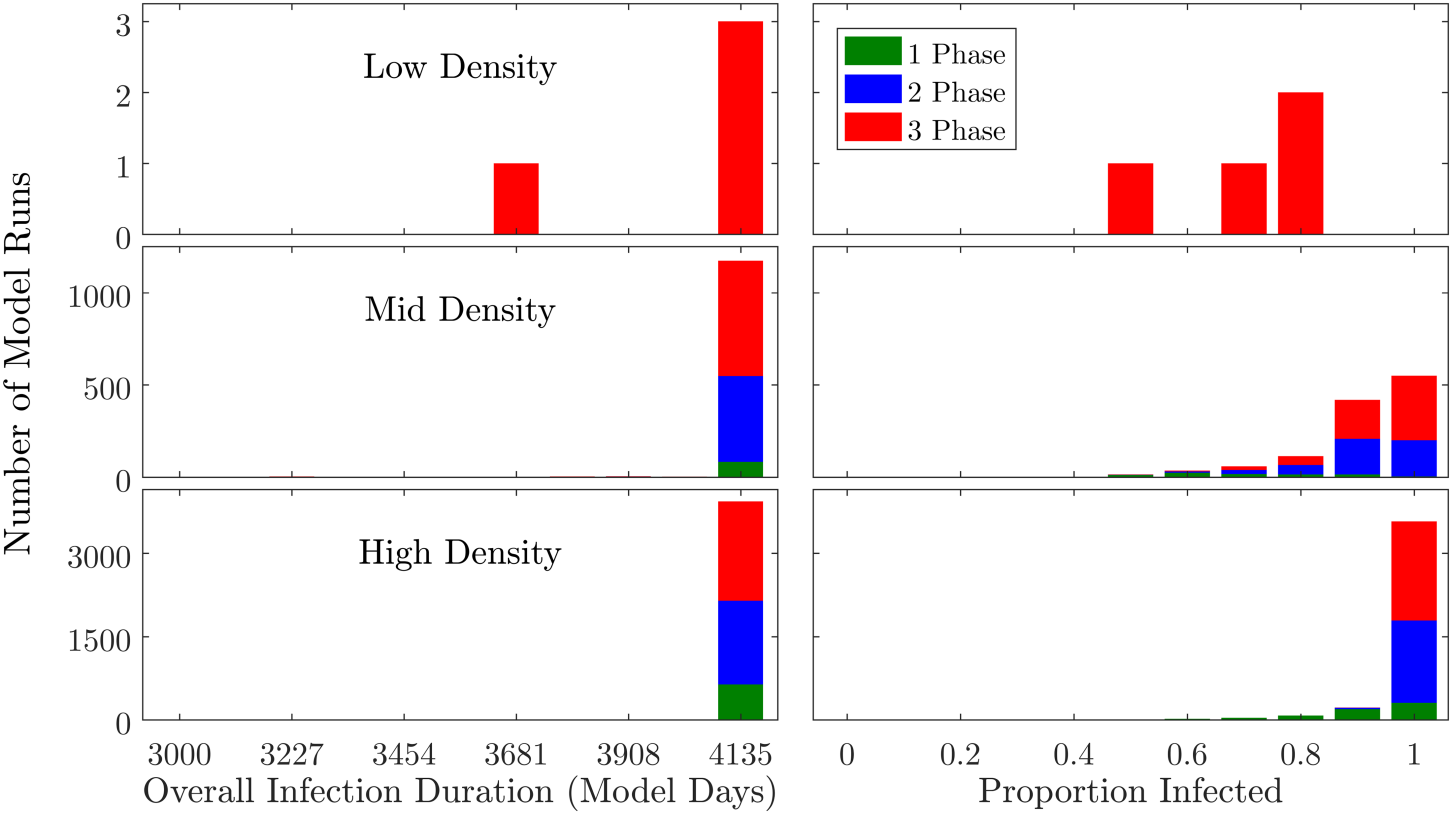
Right-censored histograms showing distribution of dependent variables. These plots are parallel to those in Fig 3, yet include only datapoints in which the infection duration was ≥ 3000 model days. Data were split into 11 bins.

Digging deeper into the behavior of the system within the subset of model runs that resulted in a long-duration “systemic” infection, we plot histograms including only runs in which the overall infection duration was ≥ 3000 model days (Fig 4). This analysis indicates that, at sufficiently-high *N*_*p*_ values, all three treatments sometimes result in full-duration (4135 model day) epidemics. Once an infection reaches the systemic level, it is very unlikely to die off naturally prior to the end of the model run. However, even among the systemic outbreaks, the scale of spreading exhibits wider variability, with the high-specialization runs more likely to result in larger epidemics.

**Fig 5:**
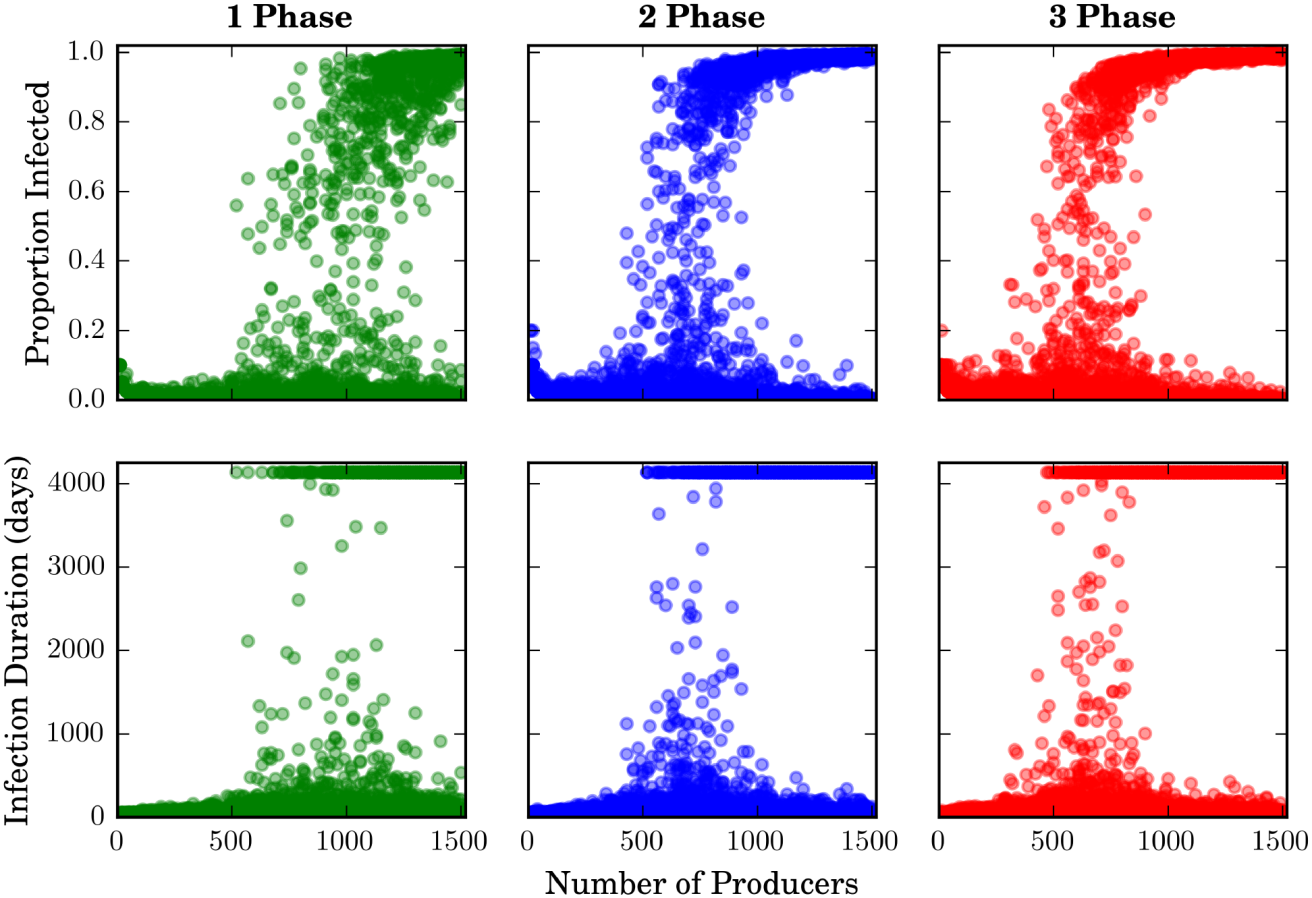
Scatter plots showing full model-output dataset for both dependent variables. Proportion of agents infected (cumulative) appears in the top row, and network-level infection duration in the bottom. Each point represents one of the 45,000 model runs (15,000 for each level of specialization).

Scatter plots of the raw data (Fig 5) reveal a nonlinearity, with *N*_*p*_ values below some value never igniting a globalized epidemic. This critical value appears to vary by specialization level. Kruskal-Wallis equality-of-populations rank tests (used due to the non-normal data distributions) indicate that the data associated with each treatment differ significantly in terms of both infection duration and proportion infected (Table 3). Based on a cursory visual analysis, for the multi-phase systems, the critical region occurs at approximately 500 ≤ *N*_*p*_ ≤ 1000, separating the unimodal phase (in which all infections are small and short) from the bimodal phase (in which infections are either small and short or large and long, but never in between). For the single-phase systems, the critical region would appear to occur around 600 ≤ *N*_*p*_ ≤ 1400. Only within the critical regions do we ever observe mid-length or mid-scale outbreaks.

**Table 3:** Kruskal-Wallis equality-of-populations rank test statistics.

Dependent Variable	Rank Sums			χ^2^	d.f.	p
	1 Phase	2 Phase	3 Phase			
Proportion infected	2.99e+08	3.47e+08	3.67e+08	962.912	2	0.0001
Infection duration	3.09e+08	3.44e+08	3.59e+08	517.021	2	0.0001

**Fig 6:**
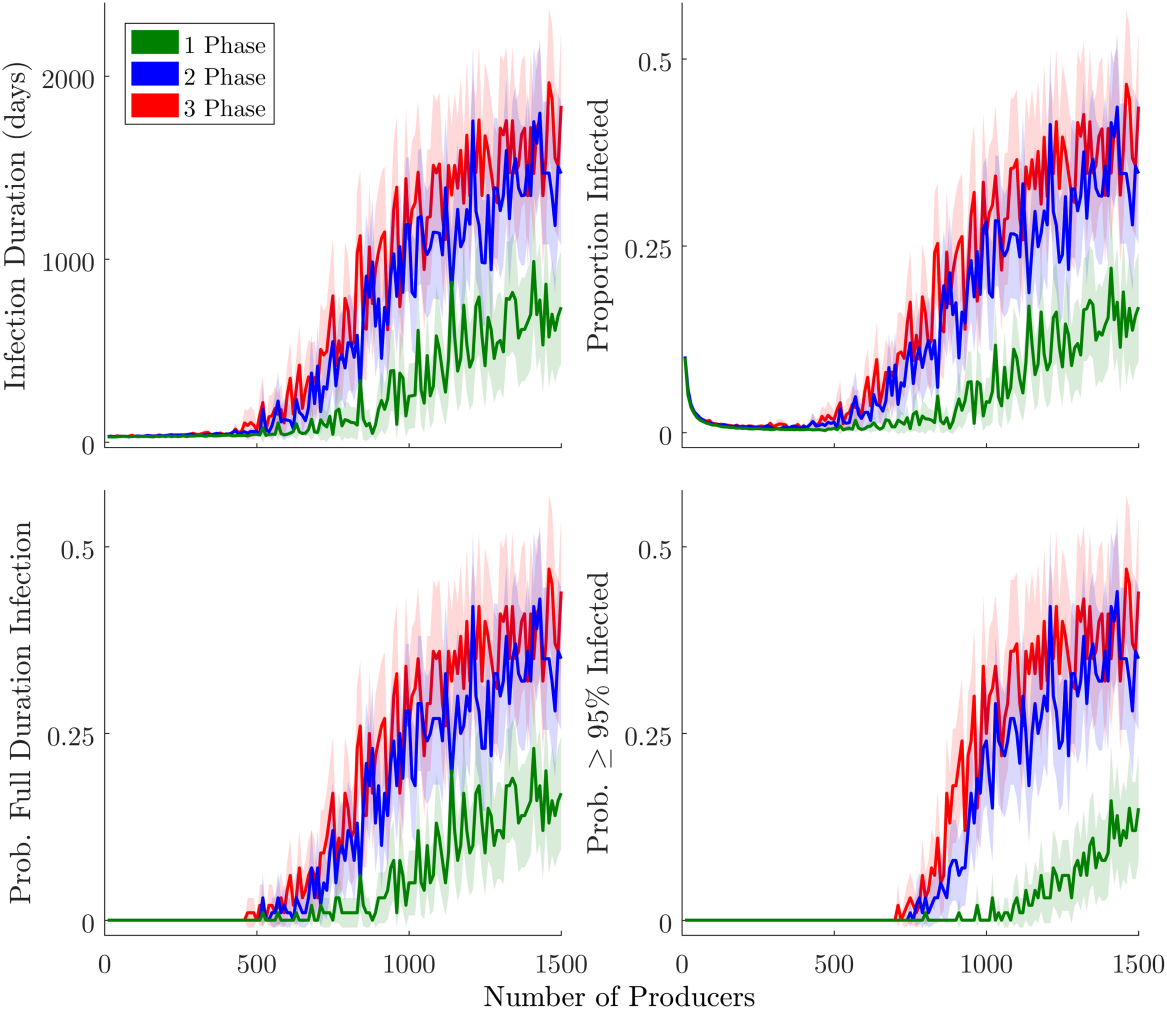
Percolation threshold visualizations. Lines plot average values for the 100 runs at each of 150 *N*_*p*_ levels, with corresponding color fields indicating 95% CI. Top left plot shows infection duration. Top right shows mean proportion infected (cumulative). Bottom left shows the fraction of runs resulting in a systemic network-level infection lasting the full duration of the model run (4135 model days). Bottom right shows the fraction of runs in which 95% or more of the agents became infected.

To investigate the apparent percolation dynamics in the raw data, we plot the mean and 95% confidence interval for both dependent variables—as well as two alternative indicators of infection severity—over the full *N*_*p*_ range (Fig 6). These data, especially the metrics that focus on large-scale and long-term infection events (bottom row), provide further evidence of a percolation threshold. We also note that variability increases dramatically as *N*_*p*_ surpasses the critical region, with higher variability in the high-specialization data.

**Fig 7:**
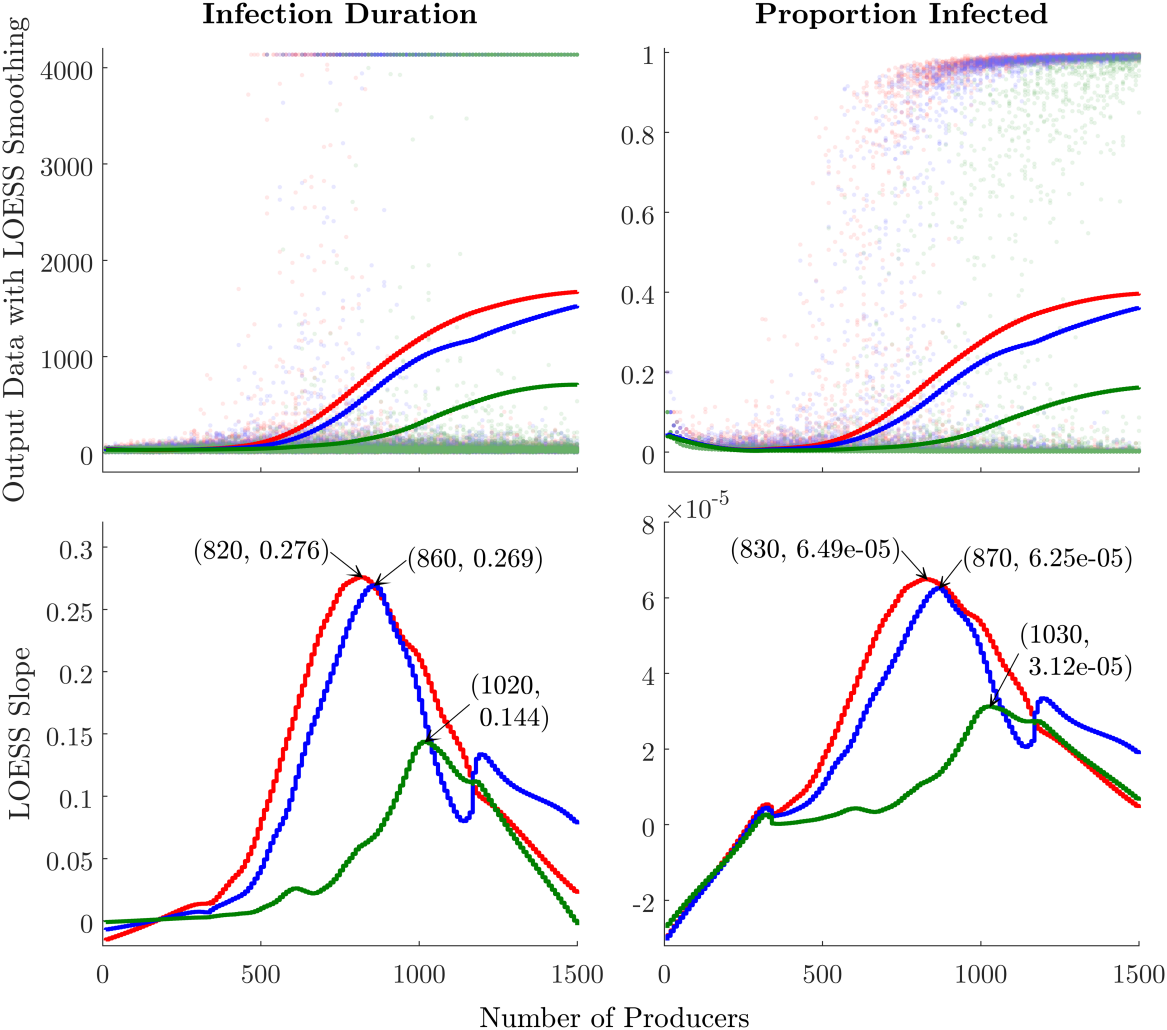
Finding percolation points numerically. Upper plots show raw model output data with LOESS-smoothed curves (span length = 0.45 × *N*). Lower plots show the slope of each LOESS curve, with maximum-slope points annotated. Green represents 1-phase, blue 2-phase, and red 3-phase treatments.

To further analyze the scaling behavior of the dependent variables, we apply LOESS smoothing to the raw model output data. The *N*_*p*_ value at which the LOESS-smoothed curve has the highest slope indicates the point at which outbreak severity scales most abruptly with *N*_*p*_, or the approximate percolation threshold. Fig 7 displays the results of this procedure, with the lower plots showing the slope of the LOESS curves as a function of *N*_*p*_. The *N*_*p*_ values corresponding to the maximum slopes (indicated in the figures) would appear to correspond to the critical *N*_*p*_ ranges observed visually in Figs 5 and 6 for all treatments. On both epidemic severity metrics, the three-phase treatment exhibits the lowest percolation threshold, as well as the highest slope at this point, indicating that the high-specialization networks are vulnerable to epidemic percolation at lower densities, and also exhibit a more-abrupt phase-change. Although the difference between the two- and three-phase systems is marginal, we can conclude that there is a marked differentiation between the behavior of single- versus multi-phase systems at criticality.

### Experiment two

#### Contact network metrics

**Fig 8:**
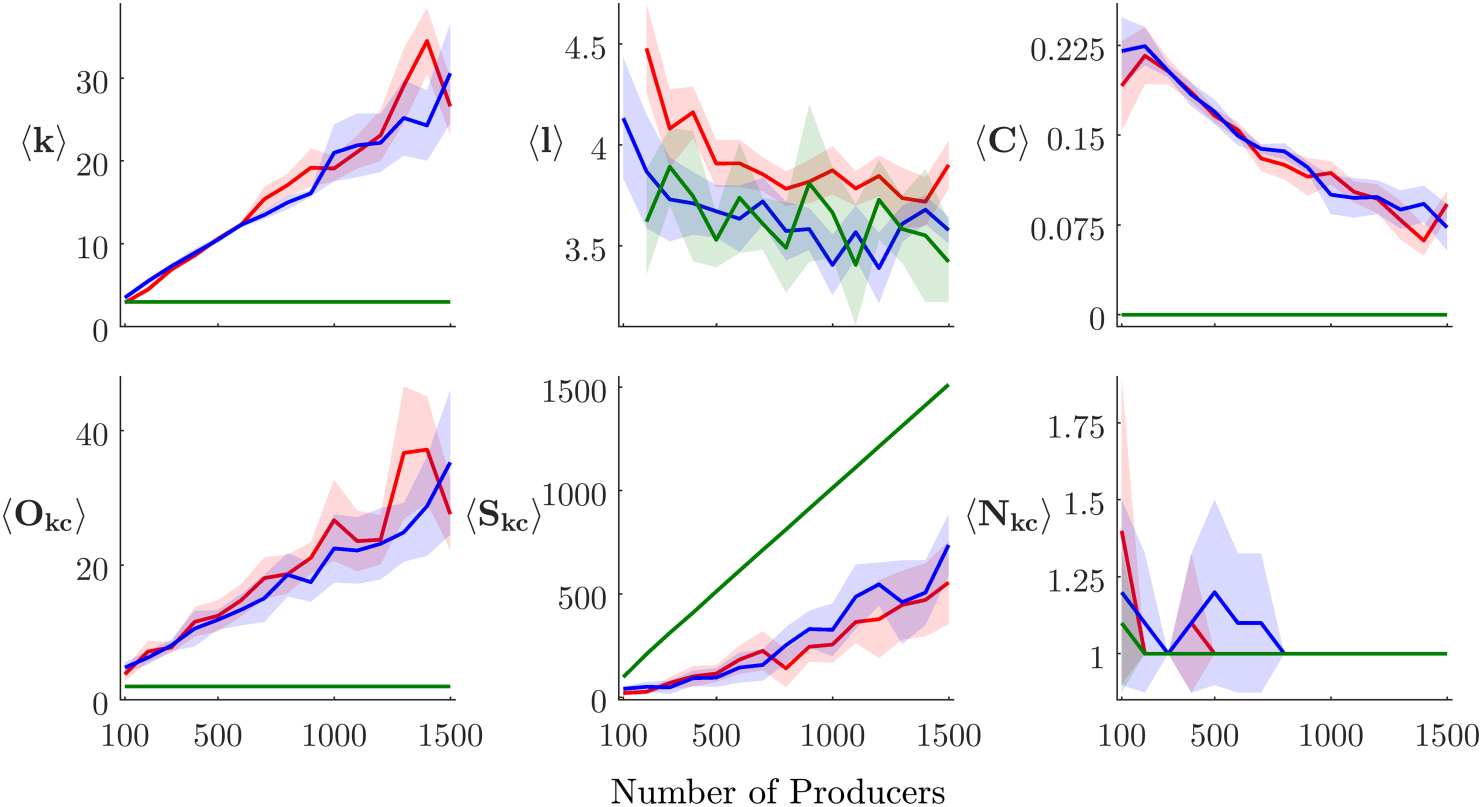
Correlating N_p_ with contact network metrics. Key contact network metrics, calculated for each treatment. Lines plot averages for each *N*_*p*_ value; color fields show 95% CI. Green represents 1-phase, blue 2-phase, and red 3-phase treatments.

**Fig 9:**
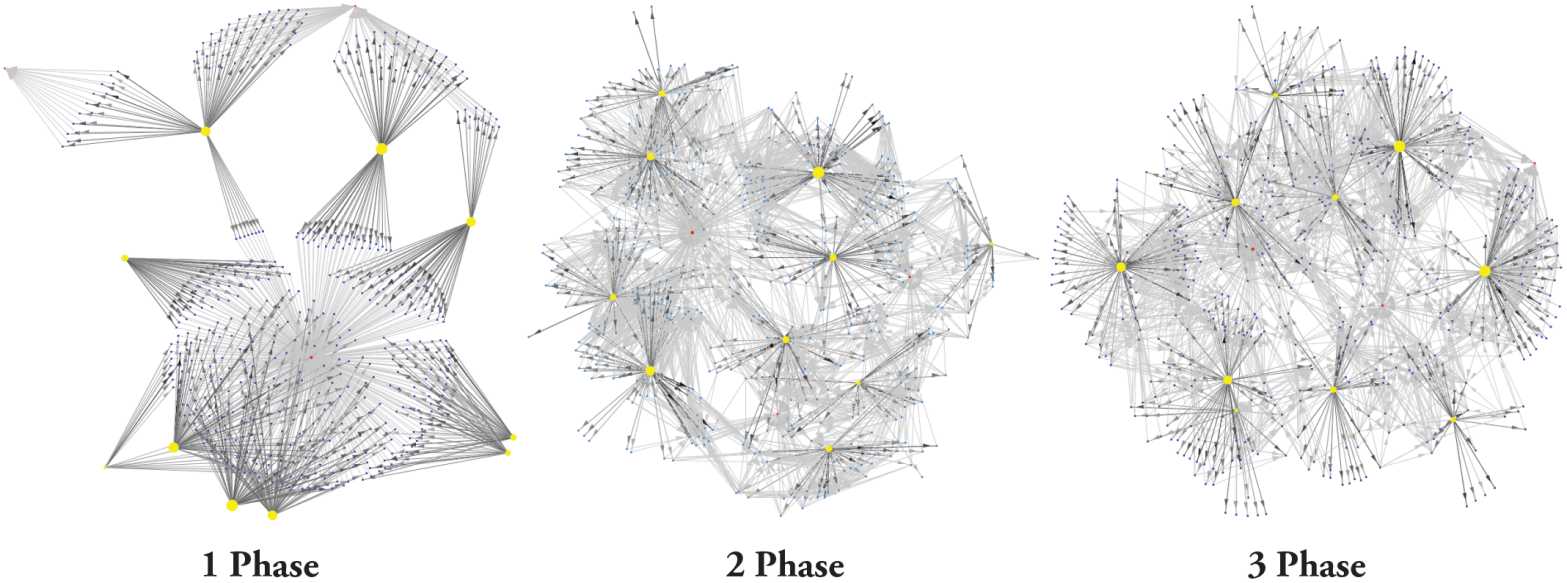
Visualizations of sample networks generated by the model under each level of producer specialization. *N*_*p*_ = 500 for each network. Nodes were positioned using a spring layout, and sized according to total number of contact events. Blue nodes are producers; yellow are feed mills, and red are slaughter plants.

Experiment one found that the greatest differences in percolation risk occur when stepping from single-phase to multi-phase systems. Here we investigate whether key network metrics may provide clues that explain this result from a network-theoretic perspective (Table 4 and Fig 8). The most striking feature in these data is that, in the single-phase networks, several of the network metrics simply do not scale with *N*_*p*_ as they do in the multi-phase scenarios. Network maps plotted from sample model runs (Fig 9) illustrate the fundamental structural difference underlying this result: in the single-phase systems, each producer is connected only to a single feed mill and a single slaughter plant. In light of this, it is clear why the average clustering coefficient 〈*C*〉—defined as the ratio of “closed triangles” to “total triangles”—will by definition be equal to zero for all single-phase runs. For the same reason, average degree 〈*k*〉 = 3, *k*-core size *S*_*kc*_ ≈ *N*_*p*_, and *k*-core order *O*_*kc*_ = 2 also hold universally.

**Table 4:** Key contact network metrics for each producer specialization level, stratified across three producer density categories.

Network Metric		0 < N_p_ ≤ 500			500 < N_p_ ≤ 1000			1000 < N_p_ ≤ 1500		
		*Mean*	*95% CI*		*Mean*	*95% CI*		*Mean*	*95% CI*	
Average Degree 〈*k*〉	**1 Phase**	3.00	3.00	3.00	3.00	3.00	3.00	3.00	3.00	3.00
	**2 Phase**	7.14	6.42	7.86	15.58	14.52	16.64	24.84	22.90	26.78
	**3 Phase**	6.70	5.90	7.50	16.62	15.67	17.57	26.88	24.98	28.78
Average Shortest Path Length 〈*l*〉	**1 Phase**	3.70	3.60	3.82	3.66	3.56	3.77	3.54	3.44	3.64
	**2 Phase**	3.82	3.72	3.92	3.58	3.52	3.64	3.57	3.51	3.62
	**3 Phase**	4.16	4.17	4.45	3.85	3.81	3.89	3.80	3.75	3.85
Clustering Coefficient *C*	**1 Phase**	0.00	0.00	0.00	0.00	0.00	0.00	0.00	0.00	0.00
	**2 Phase**	0.20	0.19	0.21	0.13	0.12	0.14	0.09	0.08	0.10
	**3 Phase**	0.19	0.18	0.20	0.13	0.12	0.13	0.09	0.08	0.09
*k*-core Order *O*_*kc*_	**1 Phase**	2.00	2.00	2.00	2.00	2.00	2.00	2.00	2.00	2.00
	**2 Phase**	8.34	7.39	9.29	17.42	15.80	19.04	26.88	23.90	29.86
	**3 Phase**	8.58	7.47	9.69	19.88	18.09	21.67	29.78	26.71	32.85
*k*-core Size *S*_*kc*_	**1 Phase**	308.40	266.63	350.17	812.76	772.16	853.36	1312.86	1272.20	1353.52
	**2 Phase**	66.12	52.13	80.11	242.42	200.33	284.51	547.80	481.56	614.04
	**3 Phase**	67.04	50.74	83.34	209.88	175.72	244.04	443.50	375.73	511.27
Number of *k*-cores *N*_*kc*_	**1 Phase**	1.02	0.98	1.06	1.00	1.00	1.00	1.00	1.00	1.00
	**2 Phase**	1.12	1.03	1.21	1.04	0.98	1.10	1.00	1.00	1.00
	**3 Phase**	1.10	1.00	1.20	1.00	1.00	1.00	1.00	1.00	1.00

For the multi-phase networks, the explanation for the higher 〈*k*〉 is trivial: an entire interaction context is added, so there must be more edges. The more important realization is that the addition of the producer–producer interaction context can add bridging edges, resulting in elevated 〈*C*〉 values. Places where these bridges connect portions of the network that would otherwise have remained isolated represent clear risk points for disease outbreaks to become systemic.

But, can any of these metrics reliably predict epidemiological risk? For the multi-phase networks, many of the metrics seem to scale roughly linearly with *N*_*p*_, with 〈*k*〉, 〈*O*_*kc*_〉, and 〈*S*_*kc*_〉 being positively correlated; and 〈*l*〉 and 〈*C*〉 being negatively correlated. Unfortunately, the lack of any significant nonlinearity suggests that the percolation point cannot be reliably predicted *a priori* by tracking these network metrics as a network grows. Developing metrics that are effective predictors of disease spread risk in complex networks remains an area for future study.

#### Infected component network metrics

**Fig 10:**
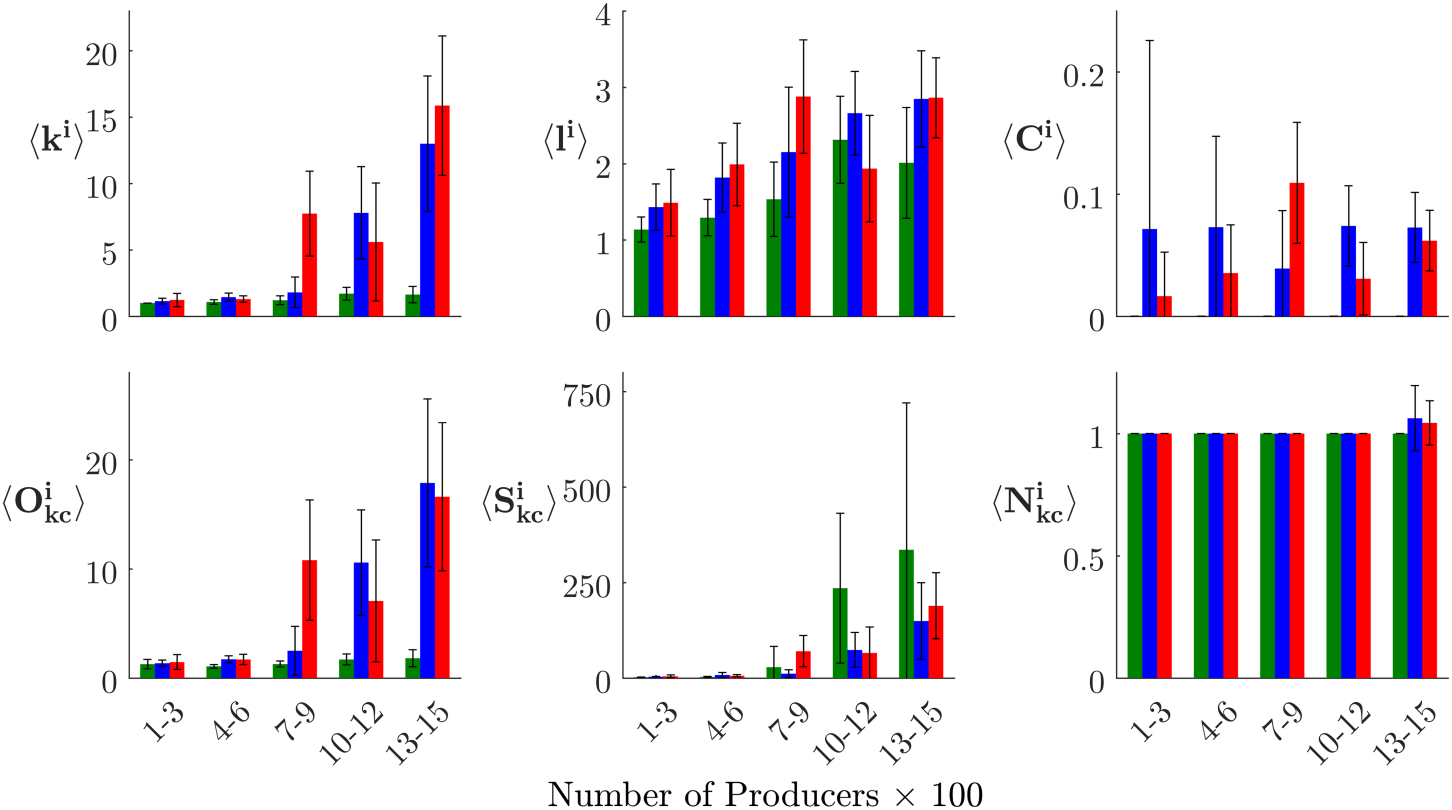
Correlating N_p_ with infected component network metrics. Key infected component network metrics, calculated for each treatment. Bars plot averages for five *N*_*p*_ ranges; whiskers show 95% CI. Green represents 1-phase, blue 2-phase, and red 3-phase treatments.

Examining metrics calculated on infected-component subgraphs (Table 5) provides insights into the network structures that underlie percolation, and how these structures differ between the single- and multi-phase networks. Overall, we note that only 226 of the 450 model runs conducted in experiment two resulted in an infection network, with the infection in the remaining 224 runs failing to spread beyond the initially-infected node. This mirrors the bimodal epidemic size distribution discussed above and shown in Figs 3 and 4. As a result of this lower *N*, *N*_*p*_ values were divided into five bins for visualization (Fig 10).

**Table 5:** Key infected component network metrics for each producer specialization level, stratified across three producer density categories.

Network Metric		0 < N_p_ ≤ 500			500 < N_p_ ≤ 1000			1000 < N_p_ ≤ 1500		
		*Mean*	*95% CI*		*Mean*	*95% CI*		*Mean*	*95% CI*	
Average Degree 〈*k*^*i*^〉	**1 Phase**	1.05	0.95	1.14	1.26	0.99	1.53	1.76	1.33	2.19
	**2 Phase**	1.25	1.08	1.42	3.95	1.70	6.21	10.64	7.10	14.18
	**3 Phase**	1.25	0.96	1.54	5.33	3.09	7.58	13.41	9.10	17.72
Average Shortest Path Length 〈*l*^*i*^〉	**1 Phase**	1.20	1.06	1.35	1.72	1.35	2.09	2.20	1.66	2.73
	**2 Phase**	1.63	1.34	1.91	2.35	1.81	2.88	2.70	2.24	3.15
	**3 Phase**	1.69	1.34	2.04	2.40	1.86	2.95	2.68	2.22	3.13
Clustering Coefficient *C*^*i*^	**1 Phase**	0.00	0.00	0.00	0.00	0.00	0.00	0.00	0.00	0.00
	**2 Phase**	0.06	-0.02	0.13	0.09	0.03	0.15	0.07	0.04	0.09
	**3 Phase**	0.02	-0.01	0.05	0.07	0.04	0.11	0.05	0.03	0.08
*k*-core Order $O_{kc}^{i}$	**1 Phase**	1.18	0.96	1.40	1.48	1.09	1.87	1.67	1.25	2.08
	**2 Phase**	1.54	1.31	1.76	5.76	2.19	9.33	14.21	9.06	19.37
	**3 Phase**	1.54	1.11	1.97	7.37	3.81	10.93	14.44	9.07	19.80
*k*-core Size $S_{kc}^{i}$	**1 Phase**	2.64	2.21	3.06	39.61	-11.78	91.00	342.62	112.69	572.55
	**2 Phase**	4.64	3.03	6.26	47.67	11.24	84.09	105.96	44.94	166.99
	**3 Phase**	4.54	2.65	6.43	50.48	21.21	79.76	158.72	90.45	226.99
Number of *k*-cores $N_{kc}^{i}$	**1 Phase**	1.00	1.00	1.00	1.00	1.00	1.00	1.00	1.00	1.00
	**2 Phase**	1.00	1.00	1.00	1.00	1.00	1.00	1.04	0.96	1.11
	**3 Phase**	1.00	1.00	1.00	1.00	1.00	1.00	1.03	0.97	1.09

**Note:** Reported values are averages for runs falling into each producer density category.

Data analyses reveal several differences between the overall contact networks and the infected component networks. Whereas 〈*C*〉 in the contact network drops as *N*_*p*_ rises, 〈*C*^*i*^〉 remains relatively flat, suggesting that subgraphs with relatively-higher clustering than the rest of a network may be especially vulnerable to disease. Secondly, in the multi-phase systems, a nonlinearity would appear to exist in 〈*k*^*i*^〉 with respect to *N*_*p*_ around the critical percolation regions. As a network grows larger, it becomes less likely that any two randomly-selected nodes will be linked, since only so many contacts can occur in a given timeframe. Therefore, heavily-connected nodes will tend to be the ones whose edges happen to impinge upon an infected trading partner. $\langle O_{kc}^{i}\rangle$ and $\langle S_{kc}^{i}\rangle$ also appear to exhibit a similar non-linearity. Interestingly, for the multi-phase networks, $\langle O_{kc}^{i}\rangle$ would seem to more heavily reflect the percolation threshold, whereas for the single-phase networks, in which it was always the case that $1<\langle O_{kc}^{i}\rangle <2$, it is $\langle S_{kc}^{i}\rangle$ that balloons upon reaching the threshold.

## Discussion

Our experimental results strongly suggest that, at least in the context of the model presented here, the risk of catastrophic infectious disease outbreaks may be inhibited by (a) sparser networks, and, perhaps more critically, (b) networks in which fewer contexts for interaction facilitate greater compartmentalization of inter-agent contact patterns, leading to both shorter and smaller outbreaks, as well as less uncertainty about whether a given outbreak will become systemic. These findings corroborate previous theoretical research into the network features that can promote large-scale epidemics [43, 44], as well as empirical studies that point to similar infection spread patterns having occurred in real-world outbreaks [2–4].

Despite the phase transitions in our data not being particularly “sharp,” there is clear evidence of a nonlinearity in the scaling of epidemic severity with producer density in the hog production systems generated by our model. Quantifying the producer densities at which adding additional producers to the system is most-strongly correlated with an increased risk of catastrophic disease spread reveals a clear differentiation between the epidemiological resilience of low- versus high-specialization treatments.

As in many dynamical systems, we find that the critical region around the percolation threshold acts as a border between a unimodal system state in which disease outbreaks virtually always die out quickly, and a bimodal state in which large-scale, systemic outbreaks are possible. It is only within the critical region that medium-severity outbreaks are observed. This finding is important because it entails that, if the size and/or duration of disease events in a growing livestock production network has begun to show wider variability, this could be an indication that further increasing the regional production density may not simply increase the risk linearly, but instead accelerate the system toward a regime characterized by the possibility for catastrophic epidemic events.

A limitation of the model concerns human behavioral adaptation in the face of epidemiological threats. Whereas the model used here assumes that agents’ behavioral heuristics remain static, previous research has pointed to the potential for behavioral adaptation—for example limiting contact as a disease becomes more prevalent—to significantly affect the course of an outbreak [73]. The extent to which such adaptive behavior may differentially-impact the disease resilience of livestock production networks with varying levels of producer specialization or spatial density remains an area for future research.

A limitation of experiment two rests in our selection of network metrics, and our choice to binarize and symmetrize the networks for analysis. While binarization and symmetrization have been employed historically in network analysis—and while there is some evidence to suggest that this approach is valuable for the evaluation of spreading dynamics [65]—future studies will compare the efficacy these metrics to their weighted and/or directed counterparts as indicators of epidemiological vulnerability. It would also be useful to analyze vulnerability not only from the whole-graph perspective, but from the level of individual nodes.

Another area for future study is to investigate percolation dynamics within mixed systems of single- and multi-phase producers. This would lend further insight to optimize risk mitigation strategies in real-world networks, which generally contain multiple overlapping production systems. For example, it would be valuable to understand the extent to which the introduction of just a few multi-phase producers into a region dominated by farrow to finish farms may impact percolation risk.

## Conclusion

Those concerned with preventing the spread of catastrophic diseases in the U.S. hog industry most-commonly promote the adoption of discrete biosecurity and biocontainment interventions at the premises level; strategies which may well be efficacious in many situations. However, epidemics are ultimately spread through complex networks of interacting actors, and—as we have shown—the structure of a given network can have a dramatic impact on the epidemiological resilience of the system. As hog production grows denser and more spatially-consolidated, it will become increasingly vital to consider how operational decisions made at the farm level impinge upon the patterns of trade and contact that may become transmission vectors in the next outbreak.

While single-phase systems may be falling out of favor for reasons of production efficiency, our results suggest that industry practitioners, managers, and regulators would be wise to consider the biosecurity advantages associated with farrow to finish farms when developing best management practices to mitigate epidemiological risk. All else being equal, systems dominated by single-phase producers should theoretically be able to withstand significantly higher farm densities without a corresponding increase in the risk of large-scale disease percolation. This is because adding a producer–producer interaction context can form bridges between otherwise-isolated parts of a network, turning what could have been a short-term, localized outbreak into an ongoing, systemic one. In hog-dense regions such as Iowa, Illinois, and North Carolina—where disease is a constant threat—a turn back toward single-phase production may offer a means to increase system-wide disease resilience, even while maintaining high regional hog production capacity.

## Supporting information

S1 Protocol RUSHPNBM v.0.8 ODD+D protocol. Describes in detail the technical specifications of the model developed for this experiment, including parameter values, calibration details, and pseudocode representations of all functions.
(PDF)

S1 Dataset Infection data. Infection duration and size data for 45,000 model runs. Used in experiment one.
(CSV)

S2 Dataset Network data. Network edge list data for all agents, across 450 model runs. Used in experiment two.
(CSV)
