# Supplementary material for: Using an agent-based model to evaluate the effect of producer specialization on the epidemiological resilience of livestock production networks
Source: PLoS One. 2018 Mar 9;13(3):e0194013. doi: 10.1371/journal.pone.0194013 (PMC5844541; doi:10.1371/journal.pone.0194013)
Supplement: S1 Protocol — Describes in detail the technical specifications of the model developed for this experiment, including parameter values, calibration details, and pseudocode representations of all functions. (PDF) [file pone.0194013.s001.pdf]

ODD+D Protocol: Regional U.S. Hog Production Network Biosecurity Model v.0.8

Serge Wiltshire, Food Systems Department, University of Vermont

9/28/2017

This material is based upon work that is supported by the National Institute of Food and Agriculture, U.S. Department of Agriculture, under award number 2015-69004-23273.

The following model description follows the ODD (Overview, Design concepts, Details) protocol for describing individual- and agent-based models (Grimm et al. 2006[1], 2010[2]), with ODD+D amendments as proposed by Müller et al. (2012[3]).

I) Overview

Purpose

The Regional U.S. Hog Production Network Biosecurity Model (RUSHPNBM), version 0.8, is an agent-based model developed to assess both supply chain network level and human-behavioral factors relevant to the spread of socioeconomically-important diseases through the U.S. hog production chain. RUSHPNBM has been developed using AnyLogic 7 software, which uses the Java programming language. Model calibration was undertaken via available statistical datasets coupled with an iterative expert informant advisory process. The model generates realistic production chain networks of producers, feed mills, and slaughter plants at the spatial scale of 431,200 square kilometers. The epidemiological spread model is of the Susceptible Infective (SI) type, with infections transmitted between agents probabilistically based on patterns of trade and contact. Disease spread probabilities associated with the different types of inter-agent contact have been calibrated by reference to epidemiological data concerning disease spread dynamics associated with previous real-world epidemic events in the hog industry, as well as input from livestock veterinarians.

The model was designed for use by university researchers, industry practitioners, veterinary specialists, and government agencies wishing to analyze the dynamics and consequences of disease spread in the U.S. hog production chain under varying assumptions concerning disease characteristics, production chain network structures, and implementation of biosecurity measures and agent behaviors that may prevent or curb catastrophic outbreaks.

---

[1] Grimm V, Berger U, Bastiansen F, Eliassen S, Ginot V, Giske J, Goss-Custard J, Grand T, Heinz SK, Huse G, Huth A, Jepsen JU, Jørgensen C, Mooij WM, Müller B, Pe'er G, Piou C, Railsback SF, Robbins AM, Robbins MM, Rossmanith E, Rüger N, Strand E, Souissi S, Stillman RA, Vabø R, Visser U, DeAngelis DL. (2006). A standard protocol for describing individual-based and agent-based models. Ecological Modelling 198:115-126.
[2] Grimm V, Berger U, DeAngelis DL, Polhill G, Giske J, Railsback SF. (2010). The ODD protocol: a review and first update. Ecological Modelling 221: 2760-2768.
[3] Müller, B., Angermueller, F., Drees, R., Dressler, G., Groeneveld, J., Klassert, C., ... & Schwarz, N. (2012). Describing Human Decisions in Agent-Based Social-Ecological Models-ODD+ D an Extension of the ODD Protocol. Available at SSRN 2044736.

Entities, State Variables, and Scales

Three classifications of hog production chain network agents, identified by industry experts as critical players in the transmission of disease, are represented in the model. These are (a) producers, (b) feed mills, and (c) slaughter plants. Producer agents are assigned one of five industry roles based on the USDA's classification system for hog producers, these being (a) Farrow to Wean, (b) Wean to Feeder (a.k.a. Nursery), (c) Feeder to Finish (a.k.a. Finish Only), (d) Farrow to Feeder, and (e) Farrow to Finish. Figure 1 below shows each agent type, its graphical representation in the model, and an outline of the heuristics that govern inter-agent contact patterns. Tables 1-2 below show the agent-level attributes and baseline parameters used in the simulations.

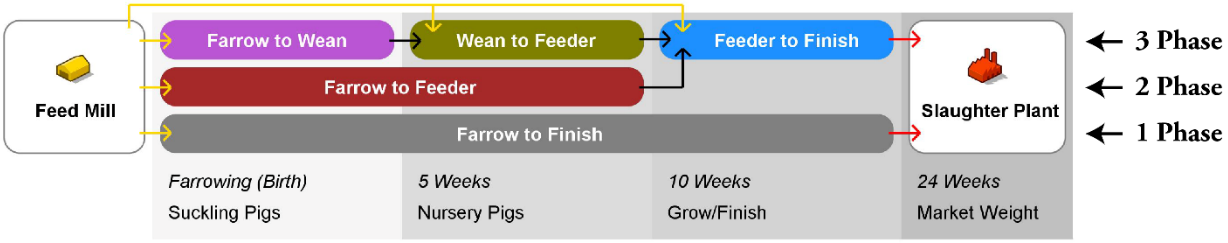

Figure 1: Structure of connections between agents, including hoofstock age transfer conditions where applicable.

Table 1.1: Parameters and variables common to all agents

Attribute	Description
Static parameters (set at initialization)	
My name	String representation encoding agent class and index (for tracking network connections)
State variables (may change throughout simulation run)	
Infectivity state	Either "clean" or "infected".
Has been infected	Flag indicating whether the agent was ever infected during a run.
Contact network out degree & in degree	List of the other agents with whom each agent had contact throughout a run, as well as the number of times contact between the two agents occurred. Out degree is incremented whenever an agent sends animals or feed; in-degree whenever animals or feed are received.
Infection network out degree	List of the other agents to whom an agent spread the disease, along with the number of times infection spread occurred between the two agents throughout a run.

Table 1.2: Parameters and variables for producer agents

Attribute	Description
Static parameters (set at initialization)	
Farm category	Encodes which of the 5 producer industry roles this agent falls into.
Total capacity	Total animal capacity.
My transferee producers	List of potential producer trading partner agent objects.
My slaughter plant	Link to slaughter plant agent object (finishing producers only).
My feed mill	Link to feed mill agent object.
State variables (may change throughout simulation run)	
Current pig inventory	Number of pigs currently on premises.
Pig batch tracker	Pigs are represented by a data structure encoding the size of each batch of stock currently making up the agent's hoofstock inventory, along with a timestamp representing that pig batch's "birthday", which is used to calculate the age of the batch.
Pig shipments in & out	Lists of sizes (number of animals) of each shipment incoming from and outgoing to the producer (used for calibration).
Feed deliveries in	Number of feed deliveries incoming to the producer (used for calibration).

Table 1.3: Parameters and variables for feed mill agents

Attribute	Description
Static parameters (set at initialization)	
My transferee producers	List of producer agent objects within service area.
State variables (may change throughout simulation run)	
Truck infected	Flag indicating whether the feed mill's delivery truck is currently infected.
Feed deliveries out	Number of outgoing feed deliveries to producers (used for calibration).

Table 1.4: Parameters and variables for slaughter plant agents

Attribute	Description
Static parameters (set at initialization)	
My transferee producers	List of producer agent objects within service area.
State variables (may change throughout simulation run)	
Pig shipments in	List of sizes (number of animals) of each shipment incoming from producers (used for calibration).

Table 2: Parameters remaining fixed throughout each model run

Parameter	Baseline Value(s)	Source
Network Makeup		
Area of network region (km2)	431,200	
Number of producer agents in the model	750	[4]
Number of livestock per producer [normal distribution, rounded to integer]	μ = 1,000; σ = 300; x ≥ 50	XE, ID, [5]
Number of producer production phases	[1, 2, 3]	
Number of slaughter plant agents in the model	3	[6]
Number of feed mill agents in the model	10	XE, ID
Epidemiological Characteristics		[7]
Suckling pig mortality rate	0.95	
Nursery pig mortality rate	0.6	
Grow/finish hog mortality rate	0.1	
Length of producer infection (days) [triangular distribution]	μ = 30; 0 ≤ x ≤ 60	
Length of slaughter plant contamination (days) [triangular distribution]	μ = 5; 0 ≤ x ≤ 10	
Farrowing		
Frequency of farrowing (days)	30	[8]
Minimum farrowing quantity as a proportion of producer capacity	0.25	XE
Producer–Producer Contact		
Maximum producer to producer connection distance (km)	100	XE, ID
Maximum frequency of pig shipments (/week)	1	XE, ID
Minimum transfer quantity as a proportion of transferee capacity	0.25	XE
Prob. of infection via trailer returning from infected transferee	0.15	XE
Feed Mill–Producer Contact		
Frequency of feed distribution trips (days)	1	XE, ID
Percent of producers in feed mill service area visited per trip	15	XE, ID
Prob. that truck will be contaminated upon visiting an infected producer	0.15	XE
Prob. that contaminated truck will infect subsequent producers on route	0.15	XE
Producer–Slaughter Plant Contact		
Prob. that infected hogs will contaminate slaughter plant receiving area	0.75	XE
Prob. of infection via truck returning from infected slaughter plant	0.15	XE

[4] FLAPS datasets, generated using tool available at: http://flaps.biology.colostate.edu/

[5] Ibid.

[6] http://usda.mannlib.cornell.edu/usda/nass/LiveSlauSu/2010s/2014/LiveSlauSu-04-21-2014.pdf

[7] https://www.aasv.org/aasv%20website/Resources/Diseases/PorcineEpidemicDiarrhea.php

[8] http://www.thepigsite.com/stockstds/3/pig-farm-targets/

Note: "XE" indicates that the value was derived using the best estimates of industry expert informants. "ID" refers to a proprietary industry dataset utilized for calibration.

The model's parameters and functions controlling pig movement and feed deliveries were further specified with the help of data provided by a Family Farm Company from the U.S. (per confidentiality, the company's name is not disclosed here). The database contains two-year records of each pig movement and each feed delivery involving producers in the Family system. The Family Farm Company consists of a network of 161 producer partners that raise pigs from birth to market. The pig movement records were used to derive realistic estimates of transfer frequencies and number of animals per transfer, as well as reinforcing USDA farm size and operational statistics (Table 2; Figure 2). The feed delivery records were used to estimate delivery frequencies (Figure 3).

Table 3: Average annual number of pig shipments per agent (producer/supplier/packer/costumer) in the Family Farm Company.

FROM	TO	N shipments FROM	N shipments TO	Avg. pigs/shipment
Wean to Finish	Wean to Finish	1.9	1.6	259
Wean to Finish	Finishing	2.5	1.7	608
Finishing	Finishing	0.5	0.5	176
Nursery	Nursery	1.2	0.8	161
Nursery	Wean to Finish	1.5	1.5	652
Nursery	Finishing	45.7	8.6	512
Supplier	Nursery	156.6	41.2	582
Supplier	Wean to Finish	60.9	10.8	406
Nursery	Customer	0.8	4	3
Finishing	Packer Customer	27.9	276.6	153
Wean to Finish	Packer Customer	22.4	179.5	153
Nursery	Packer Customer	0.5	1	2

Note: The columns list the following information. "FROM": the type of shipping agent (producer/supplier). "TO": receiving agent (producer/packer/costumer). "N shipments FROM": average annual shipments per shipping agent. "N shipments TO": average annual shipments per receiving agent. "Avg. pigs/shipment": average number of pigs per shipment.

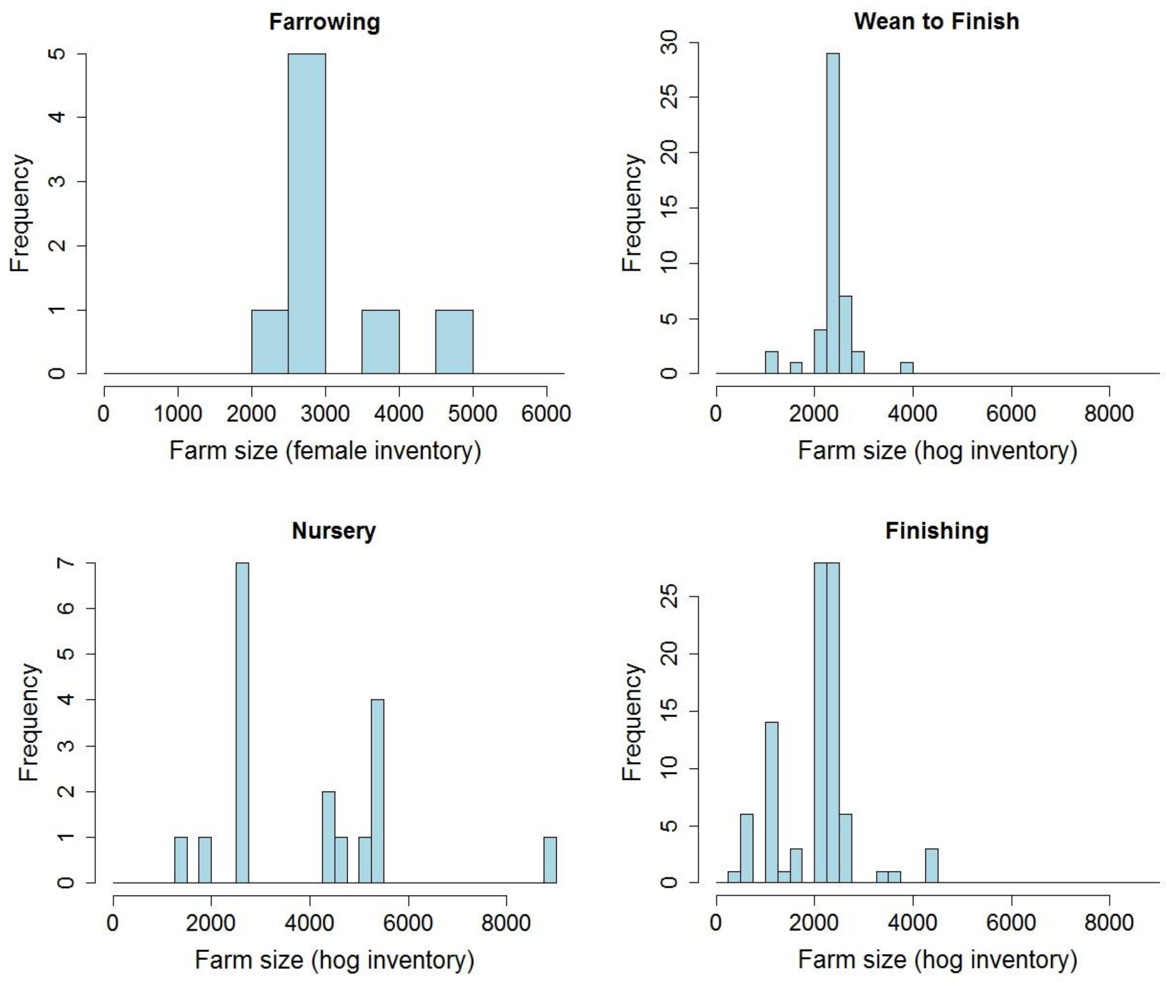

Figure 2: Farm size distribution by farm type (farrowing, wean-to-finish, nursery, finishing) in the Family Farm Company.

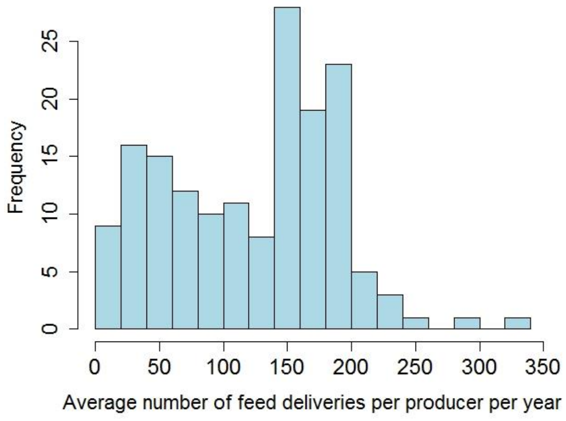

Figure 3: Average number of feed deliveries per producer per year in the system of Family Farm Company.

Finally, a team of experts in veterinary medicine and in agent-based modeling has followed the development of the model and collaborated in parametrizing, calibrating and ground truthing it:

- Julie Smith (DVM, PhD, https://asci.uvm.edu/?Page=faculty/smith/homepage.html)
- Steve Dritz (DVM, PhD, Swine Specialist, https://www.vet.k-state.edu/education/dmp/faculty-staff/faculty/dritz/)
- Asim Zia (PhD, http://www.uvm.edu/~azia/)
- Christopher Koliba (PhD, http://www.uvm.edu/~ckoliba/index.htm)

How is space included in the model?

The model is spatially situated in a continuous, two-dimensional environment representing 880 x 490 km. Distances between agents are calculated "as the crow flies." In some cases, distance is a factor in determining inter-agent contact patterns (detailed below).

How is time represented in the model?

The model's time scale is based on real-world days, with the initial model date set to January 1st, 2012. 2012 was chosen because FLAPS initialization data are drawn from the 2012 USDA Census of Agriculture. The model's stop date can be set as desired depending on the experimental phenomena the user is interested in studying, with a default setting of January 1st, 2022.

<u>Process Overview and Scheduling</u>

All event scheduling in the model follows a Last-In-First-Out (LIFO) protocol. Four classes of functions define the operation of the model, in order of the point(s) in the simulation that they occur. First are the initialization functions, which define how the agents will be physically situated in the space, set each agent's individual parameters, and identify lists of potential trading partners based on the classification and industry role of the agent, as well as spatial proximity to other agents. Second are the cyclically-executing functions, which make up the agents' decision rules, determining how and when contact between agents will occur (through the transfer of livestock and the distribution of feed), and thereby opening potentials for infection to spread. These functions also determine and implement the consequences of an infection upon the agent. Third is the initial infection function, which occurs after the initial transient period. Finally, fourth are the set of functions facilitating the output of model data for further analysis, including post-experiment scripts to parse model outputs and analyze results across multiple runs.

II) Design Concepts

Theoretical and Empirical Background

Because real-world epidemics are fundamentally phenomena which propagate through networks (social, business, transportation, etc.), the formulation of a suitably-realistic network structure within which agents operate is a fundamental basic principle of the model. A corollary to this basic principle concerns the model's balance between context specificity and analytic transparency. The model's network generation algorithm strives to maintain sufficient context specificity to capture the critical complexities underpinning observed epidemiological spread phenomena, while bracketing superfluous elements of real-world production chain networks that have not been implicated in previous epidemiological events. For example, the model contains only feed mill, producer, and slaughter plant agent typologies, because these were identified by industry experts as the critical players underpinning disease spread. Whereas in real-world hog production chain networks there may be a multitude of other actor typologies (i.e. auction houses, equipment suppliers, construction contractors, insurance agents, and many more), these were intentionally excluded from the model's design to simplify analysis.

Another guiding principle is the spatial framework of the model. In many epidemiological studies, agent density has been shown to impinge directly upon spread characteristics. With high enough density, complex phenomena such as percolation thresholds may emerge. To study this, the model was designed with the ability to flexibly change the density of agents in space.

On what assumptions is/are the agents' decision model(s) based?

The primary assumptions driving agent behavior relate to trade patterns associated with the industry role each agent plays. For example, it is assumed that, as soon as their livestock batches reach the transfer age appropriate for their industry role, producer agents will search the agent space for appropriate trading partners.

Another assumption concerns the characteristic distance over which agents may interact. This can be adjusted by tuning the maximum connection distance parameter. Since specific spatial location data were not available in either the USDA statistics or the Family Farm System dataset, baseline values were estimated in consultation with industry experts.

Learning

The agents' decision rules remain non-adaptive at this stage of model development. The agents' action heuristics are based on their industry roles, and are designed to realistically replicate throughput in the production chain system as a whole. Thus, an agent will transfer hoofstock to an appropriate trading partner as soon as possible, farrowing will proceed regularly wherever a producer has sufficient excess capacity, and feed deliveries will take place at a set frequency. The agents' behavior does not change as a result of model conditions, for example the presence of a disease within the network, however each agent will necessarily adapt to conditions resulting from the factors such as the number of other agents in the space, or the currently-available spare capacity of its trading partners. In future model versions, adaptive agent behavior will be implemented to reflect the decision-making heuristics of real-world hog producers, as identified through data gathering efforts presently underway.

Collectives

Livestock in the model may be considered as collectives, as they are encoded in batches of animals of the same age, and with the same infectivity status. If a producer is infected, it is assumed that all livestock on the premises become infected. This simplifying assumption follows from veterinary reports on the virility of PEDv, which tends to quickly sweep through entire herds. From a standpoint of practicality, encoding livestock in this manner was also desirable because it significantly reduces the computational time required for each run.

In addition, while not defined explicitly as such, groups of agents in the model exhibit emergent collective characteristics due to their distribution within the model's spatial framework. For example, in densely-packed areas, groups of agents tend to interact heavily within connected clusters, leading to localized disease outbreaks. This type of collective behavior is not directly imposed, but rather emerges from interactions between the model parameterization and agents' fixed behavioral rules.

Heterogeneity

As described in the *Entities, state variables, and scales* section above, agents fall into three main categories: (a) producers, (b) slaughter plants, and (c) feed mills. Producer agents are assigned one of five industry roles based on the USDA classification system for hog producers. An agent's industry role determines the initial age of its hoofstock, its hoofstock age transfer condition, as well as its set of potential trading partners. These relationships are visualized in Figure 1. Agents' decision-making heuristics also vary according to their class. For example, a farrow-to-wean producer will only send pigs to wean-to-feeder producers.

Stochasticity

RUSHPNBM uses stochasticity for initialization of agent locations and parameters, as well as for controlling infection spread. A random seed is used, such that all runs are different. These stochastic features ensure that the contact patterns that unfold in each model run are never repeated.

Draws from distribution functions (normal, uniform, and triangular) are utilized in some cases. For example, the age of the pig groups initially associated with each producer are drawn from a uniform distribution bounded according to the producer's industry role. Triangular distributions underlie the time agents will remain infected before transitioning back to the susceptible state.

Stochasticity is also used in all disease-spread events. Uniform probability distributions returning "true" if a randomly-drawn value between zero and one is less than p are used to determine if the infection will spread whenever contact between a susceptible and an infected agent occurs. Different probability values are used for each mode of transmission.

Observation

The model tracks in real-time the current hoofstock inventory of all producers in the model, the number of currently infected hoofstock, the number of currently infected producers, and the cumulative number of

infected producers, which can be output as time-series data to examine infection-spread dynamics. The total infection duration is also recorded.

In addition, we track the flow of feed and livestock between different types of agents in order to calibrate model parameters to reflect real-world data, for example the distribution of hog shipment sizes and delivery frequencies characteristic of real hog supply chain networks.

Finally, a contact network adjacency matrix with link weights encoding the number of times each agent interacted throughout the model run is exported as tabular data after each run, and later parsed using a series of Python functions. An infection-spreading network is similarly tracked, output, and parsed. Key statistics on trade and infectivity patterns across a series of model runs—both at the individual agent as well as the whole-network level—may then be analyzed.

Emergence

Emergent phenomena in the present model occur as a result of the interaction between agents' behavioral heuristics and structural elements of the model, for example the network configuration and disease spread characteristics specified by the user. This could take the form of differential spread characteristics—such as in the observation of percolation thresholds—resulting from user-input parameters concerning network makeup, probabilities, or duration parameters.

III) Details

Implementation Details

The model was implemented using AnyLogic version 7 software, which relies upon the Java programming language for all scripts and functions. The sections below use pseudocode to describe in detail the algorithmic structures underlying each model function.

Notes on pseudocode used in this document:

- The characters "//" will be used to designate a comment (i.e., the line of text following the "//" is not part of the actual function logic).
- Parameters referenced in all functions refer to those associated with the agent object from which a function has been called. In some cases, to disambiguate, the terms "self" or "my" may be used to refer to the function-calling agent object or its associated parameters.
- "`ADD OR INCREMENT [sender] in [receiver]'s [network edge list]`" is defined here as:

```
IF [sender] is not in [receiver]'s [network edge list] ADD [sender] to [receiver]'s
       [network edge list] with contact counter set to 1
ELSE INCREMENT contact counter associated with [sender] in [receiver]'s [network edge
       list] by 1
```

- "`RANDOM DRAW using [probability]`" is defined here as the Boolean value resulting from:

```
(DRAW random number from uniform distribution between 0 and 1) < [probability]
```

Is the model accessible, and if so where?

While the raw source code for the model is not accessible, the pseudocode below may be used as a guide to understand the model's structure and logic with a high level of detail.

<u>Initialization</u>

The model is initialized by progressing through a series of initialization functions. Agent parameters such as location are set as each agent object is generated by the model. Next, producer agents initialize their operational parameters and define their networks of potential trading partners to be referenced throughout the model run.

- Initialize agent locations:

All agents are placed at a random location in the continuous 2D space.

```
FOR EACH agent object
   SET x coordinate to RANDOM INTEGER between 0 and 880
   SET y coordinate to RANDOM INTEGER between 0 and 490
```

- Producer agent initialize category function:

The initialize category function sets the industry role of producers according to the specialization level to be evaluated.

```
IF (specialization level = "low")
   SET farm category to "farrow to finish"
ELSE IF (specialization level = "medium")
   SET farm category to RANDOM DRAW from ["farrow to feeder", "feeder to finish"]
ELSE IF (specialization level = "high")
   SET farm category to RANDOM DRAW from ["farrow to wean", "wean to feeder",
         "feeder to finish"]
```

- Producer agent initialize farm function:

The initialize farm function sets the producer's maximum capacity and adds one pig batch equal to this capacity to the pig batch tracker, with an age corresponding to the producer's industry role.

```
SET total capacity to MAX of 50 and (ROUND to INTEGER (DRAW from normal distribution
      with μ = 1,000 and σ = 300))
ADD pig batch to pig batch tracker with size equal to total capacity AND birthday
      equal to to a random integer between the maximum and minimum age of a pig for
      the agent's industry role
SET current inventory to total capacity
```

- Producer agent initialize network function:

Once the agents' locations and operational parameters have been initialized, a network initialization function generates a set of potential trading partners. All producer agents are assigned to the nearest feed mill, and finishing producers are also assigned to the nearest slaughter plant. A pool of potential transferee producers is also generated for each non-finishing producer according to their industry role. These relationships are shown in Figure 1. The potential transferee producers in this pool are limited by the maximum producer-to-producer connection distance parameter.

```
SET potential farms list to (SORT by distance (FILTER other producer agents s.t.
       (industry role of other producer is the next step in the production chain) AND
       (distance to the other producer <= max producer-producer connection distance
       global parameter)))
IF industry role is a finishing type
   SET my slaughter plant to closest slaughter plant
SET my feed mill to closest feed mill
ADD self to my feed mill's "links to farms" list
```

Is the initialization always the same, or is it allowed to vary among simulations?

The initialization of the spatial location, operational characteristics, and potential trading partners for each agent, and initial livestock ages differ between runs. However, the distributions of from which these values are drawn, as well as the basic heuristics controlling the behavior of each type of agent, do not change.

Are the initial values chosen arbitrarily or based on data?

Initialization parameters rely upon several datasets, including the University of Colorado / USDA FLAPS system, USDA NASS data, USDA APHIS data, Google Maps queries, proprietary industry datasets, and expert input. For details, see the Entities, State Variables, and Scales section above.

<u>Submodels</u>

- Producer agent cyclically-executing functions:

Farrow, wean, and batch piglets function:

If a farm that farrows piglets (Farrow to Wean, Farrow to Feeder, or Farrow to Finish types) is left with excess capacity after a livestock transfer, a periodic farrowing function fills that capacity with a new batch of piglets, whose birthday is set to the current model day. Once again, to eliminate unrealistically-small pig groups, a minimum farrowing size as a proportion of the farrowing farm's total capacity is required for the farrowing function to proceed. Thus, a farm which is already almost at maximum capacity will not farrow a new batch of piglets until another batch has been shipped to an appropriate trading partner.

```
**Recurrence time is the frequency of farrowing global parameter**
IF industry role is a farrowing type
   Number to wean and batch = remaining pig capacity
```

```
IF (number to wean and batch >= (minimum farrowing batch proportion * my
        capacity))
    ADD number to wean and batch and birthday = current day to pig batch tracker
    INCREMENT pig inventory by batch size
```

Ship to transferee farms function:

Non-finishing producers transfer hoofstock to a transferee farm when the hoofstock reach the age corresponding to the transfer condition associated with the industry role of the producer. This function periodically evaluates whether the transfer age requirement of a pig batch has been met. If so, the producers in the transferring producer's pool of possible trading partners are sequentially evaluated to determine whether they are able to receive the shipment. To eliminate the transfer of unrealistically-small groups of livestock, transfers will only proceed if the pig batch size exceeds the minimum transfer quantity, as a proportion of the transferee's total capacity.

If the transferring producer is infected but the transferee is not, the transferred hoofstock will automatically spread the infection to the transferee producer. If the transferee producer is infected but the transferring producer is not, the "delivery trailer" returning from the infected transferee producer may infect the transferring producer according to a probability set at model initialization.

The birthday parameter associated with the batch of transferred stock is maintained as it is passed to the transferee, such that the pig batch will once again be appropriately transferred to the next production phase at the correct transfer age. In the rare case that a pig batch exceeds the slaughtering age before a suitable transferee producer could be located, it is culled to make room for a new batch of pigs.

```
**Recurrence time is the maximum frequency of pig shipments global parameter**
IF industry role is NOT a finishing type
    FOR EACH pig batch meeting age transfer requirement
        FOR EACH transferee in my transferee producers
            IF (batch size <= transferee's spare capacity) AND (batch size >=
                    transferee's minimum batch size)
                IF (transferee's infectivity state is "infected") AND (infectivity state
                        is "clean")
                    DECREMENT batch size according to mortality rate global parameter
                            associated with pigs' age
                REMOVE pig batch from pig batch tracker
                DECREMENT pig inventory by batch size
                ADD pig batch and birthday to transferee's pig batch tracker
                INCREMENT transferee's pig inventory by batch size

                // update contact network trackers
                ADD OR INCREMENT transferee in contact network out-degree list
                ADD OR INCREMENT self in transferee's contact network in-degree list

                // update pig shipment trackers
                ADD batch size to pig shipments out list
```

```
            ADD batch size to transferee’s pig shipments in list

            // infection brought to transferee via infected pigs
            IF infectivity state is “infected”
               SET transferee’s infectivity state to “infected”
               ADD OR INCREMENT transferee in infection-spreading network out
                     degree list

            // infection brought home via trailer from transferee farm
            IF (transferee’s infectivity state is “infected”) AND (RANDOM DRAW using
                  Prob. pig truck will become contaminated if producer is infected)
                  AND (RANDOM DRAW using Prob. producer will become infected if
                  returning pig truck is contaminated)
               SET infectivity state to “infected”
               ADD OR INCREMENT self in transferee’s infection-spreading network
                     out degree list

   // cull pigs that are too old and were never able to be transferred
   FOR EACH pig batch over 168 days old REMOVE batch from pig batch tracker
```

Ship to slaughter plant function:

Finishing producers (Feeder to Finish and Farrow to Finish types) ship hoofstock to either an auction house (as described above), or directly to their slaughter plant, as soon as the hoofstock reach the designated slaughtering age. If the transferring producer is infected, the receiving area of the slaughter plant may become contaminated according to a probability set at model initialization. If the receiving area of the slaughter plant is already contaminated, the “delivery trailer” returning to the transferring producer may carry the infection back to that producer according to another probability set at model initialization.

```
IF industry role is a finishing type
   FOR EACH pig batch meeting age transfer requirement
      REMOVE pig batch from pig batch tracker
      DECREMENT pig inventory by batch size

      // update contact network trackers
      ADD OR INCREMENT slaughter plant in contact network out-degree list
      ADD OR INCREMENT self in slaughter plant’s contact network in-degree list

      // update pig shipment trackers
      ADD batch size to pig shipments out list
      ADD batch size to slaughter plant’s pig shipments in list

      // infection brought to slaughter plant via infected pigs
```

```
IF (infectivity state is “infected”) AND (RANDOM DRAW using Prob. slaughter
        plant receiving area will become infected if pig batch is infected)
    SET slaughter plant’s infectivity state to “infected”
    ADD OR INCREMENT slaughter plant in infection-spreading network out degree
            list

// infection brought home via trailer from slaughter plant
IF (slaughter plant’s infectivity state is “infected”) AND (RANDOM DRAW using
        prob. pig truck will become contaminated if receiving area is infected)
        AND (RANDOM DRAW using prob. producer will become infected if returning
        pig truck is contaminated)
    SET infectivity state to “infected”
    ADD OR INCREMENT self in slaughter plant’s infection-spreading network out
            degree list
```

- Feed mill agent cyclically-executing functions

Feed mills periodically generate delivery routes encompassing a subset of producers within their latent feed-mill-to-producer link set. Each route encompasses a subset of the producers in the feed mill’s service area, with the number of stops in each trip resulting from a draw from a Poisson distribution. While there is no actual “feed truck” object in the model, the logic of the following function is based on the way such a truck would move between agents and possibly spread disease.

Beginning from the mill, this conceptual feed truck will visit the previously-drawn number of randomly-selected producers within the feed mill’s service area before finally returning to the feed mill. If the feed mill is infected, the truck may be contaminated initially. Should the truck encounter an infected producer on its route, it may become contaminated at that point. Once a truck is contaminated, the infection may be spread to subsequent producers on the route. If a contaminated truck returns to the feed mill, the mill itself may become infected.

Distribute feed function:

```
**Recurrence time is the frequency of feed deliveries global parameter**
// generate delivery route
FOR number of producers in service area * percent of producers in feed mill service
        area visited per trip
    ADD random producer in service area (that is not already in delivery route list)
            to delivery route list

// parse infectivity consequences of delivery route
FOR EACH producer in delivery route list
    // update contact network trackers
    ADD OR INCREMENT producer in contact network out-degree list
    ADD OR INCREMENT self in producer’s contact network in-degree list

    //infected truck infects farm it's delivering to
```

```
IF (truck infected is true) AND (RANDOM DRAW using prob. producer will become
      infected if feed truck is contaminated)
   SET producer’s infectivity state to “infected”
   ADD OR INCREMENT producer in infection-spreading network out degree list

//truck becomes infected from delivery to infected farm
IF (producer’s infectivity state is “infected”) AND (RANDOM DRAW using prob. feed
      truck will become contaminated if producer is infected)
   SET truck infected to “true”
```

- Initial infection function

The system is initialized with all agents free of infection. After one model year has passed, an infection is introduced to a random producer agent. The reason for the one-year lag is to skip the transient period and allow the model to stabilize before analyzing the effect of an introduced disease. This lag is necessary because, as in a real production chain, a certain amount of slack, or a difference between the theoretical production capacity and actual production, is characteristic in the modeled production chains. In the model, this economic slack is due to the producers sometimes temporarily operating at less than maximum hoofstock capacity until an appropriate shipment of livestock becomes available. In general, after about 9 months, the level of slack in the model has stabilized.

```
**Function is called only once, after one model year**
SET one randomly chosen producer agent’s infectivity state to “infected”
```

- Infection control functions

Susceptible/infective state charts:

Each agent has an embedded state chart which encodes its infectivity status, with clean and infected states corresponding to the classical susceptible/infective framework. Should an agent become infected, a function is called which calculates the number of its stock that will die of the disease. The proportion of livestock that succumb to the disease is based on their age, with uniform mortality rates set at model initialization for suckling pigs, nursery pigs, and grow/finish hogs. After die-off is calculated for pig batches of each life stage within an infected producer’s inventory, the producer’s inventory data are updated accordingly. An agent will remain infected for a duration drawn from a triangular distribution whose mean length in days is controlled by parameters specific to each agent type.

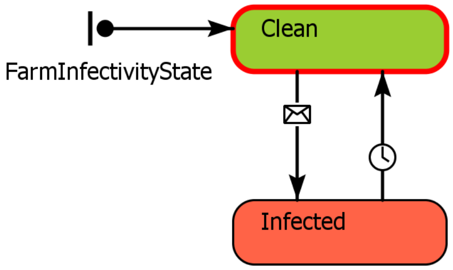

Figure X: Infectivity State Chart

Calculate mortality function:

```
FOR EACH pig batch in pig batch tracker
   DECREMENT pig batch size by global parameter encoding mortality proportion
         appropriate for age of pigs
```

- Data output

At the conclusion of each run, the model determines whether a parameter variation experiment is being conducted, and, if so, a line is added to an output csv file containing the number of producers in that run, the total infection duration, the proportion of agents that had been infected, and the specialization level.

If desired, a contact network adjacency matrix with link weights encoding the number of times each agent interacted throughout the model run, as well as the specialization level and repetition number, is generated. This is accomplished by looping through all agents and adding a line to a csv file for each entry in the agent's contact tracker. Subsequent network analysis may then be performed using external scripts.

Finally, for calibration purposes, it is possible to output the flow of feed and livestock between all agents throughout the run. A csv is generated that encodes the size and date of each transfer, as well as the IDs of the sending and receiving agents. Agent parameters such as classification and operational details are exported to another csv. The resulting data may then be compared to real-world data, and parameters tuned so that agents' actions in the model more closely mirror the distribution of hog shipment sizes and delivery frequencies observed in real hog supply chain networks.
